# Longitudinal Trends in Childhood Insulin Levels and Body Mass Index and Associations With Risks of Psychosis and Depression in Young Adults

**DOI:** 10.1001/jamapsychiatry.2020.4180

**Published:** 2021-01-13

**Authors:** Benjamin I. Perry, Jan Stochl, Rachel Upthegrove, Stan Zammit, Nick Wareham, Claudia Langenberg, Eleanor Winpenny, David Dunger, Peter B. Jones, Golam M. Khandaker

**Affiliations:** 1 Department of Psychiatry, University of Cambridge School of Clinical Medicine, Cambridge, United Kingdom; 2 Cambridgeshire and Peterborough NHS Foundation Trust, Cambridge, United Kingdom; 3 Department of Kinanthropology, Charles University, Prague, Czechia; 4 Institute for Mental Health, University of Birmingham, Birmingham, United Kingdom; 5 Centre for Academic Mental Health, Population Health Sciences, Bristol Medical School, University of Bristol, Bristol, United Kingdom; 6 MRC Centre for Neuropsychiatric Genetics and Genomics, Cardiff University, Cardiff, United Kingdom; 7 MRC Epidemiology Unit, University of Cambridge School of Clinical Medicine, Cambridge, United Kingdom; 8 Department of Paediatrics, University of Cambridge School of Clinical Medicine, Cambridge, United Kingdom; 9 MRC Integrative Epidemiology Unit, Population Health Sciences, Bristol Medical School, University of Bristol, Bristol, United Kingdom

## Abstract

**Importance:**

Cardiometabolic disorders often occur concomitantly with psychosis and depression, contribute to high mortality rates, and are detectable from the onset of the psychiatric disorders. However, it is unclear whether longitudinal trends in cardiometabolic traits from childhood are associated with risks for adult psychosis and depression.

**Objective:**

To examine whether specific developmental trajectories of fasting insulin (FI) levels and body mass index (BMI) from early childhood were longitudinally associated with psychosis and depression in young adults.

**Design, Setting, and Participants:**

A cohort study from the Avon Longitudinal Study of Parents and Children, a prospective study including a population-representative British cohort of 14 975 individuals, was conducted using data from participants aged 1 to 24 years. Body mass index and FI level data were used for growth mixture modeling to delineate developmental trajectories, and associations with psychosis and depression were assessed. The study was conducted between July 15, 2019, and March 24, 2020.

**Exposures:**

Fasting insulin levels were measured at 9, 15, 18, and 24 years, and BMI was measured at 1, 2, 3, 4, 7, 9, 10, 11, 12, 15, 18, and 24 years. Data on sex, race/ethnicity, paternal social class, childhood emotional and behavioral problems, and cumulative scores of sleep problems, average calorie intake, physical activity, smoking, and alcohol and substance use in childhood and adolescence were examined as potential confounders.

**Main Outcomes and Measures:**

Psychosis risk (definite psychotic experiences, psychotic disorder, at-risk mental state status, and negative symptom score) depression risk (measured using the computerized Clinical Interview Schedule–Revised) were assessed at 24 years.

**Results:**

From data available on 5790 participants (3132 [54.1%] female) for FI levels and data available on 10 463 participants (5336 [51.0%] female) for BMI, 3 distinct trajectories for FI levels and 5 distinct trajectories for BMI were noted, all of which were differentiated by mid-childhood. The persistently high FI level trajectory was associated with a psychosis at-risk mental state (adjusted odds ratio [aOR], 5.01; 95% CI, 1.76-13.19) and psychotic disorder (aOR, 3.22; 95% CI, 1.29-8.02) but not depression (aOR, 1.38; 95% CI, 0.75-2.54). A puberty-onset major increase in BMI was associated with depression (aOR, 4.46; 95% CI, 2.38-9.87) but not psychosis (aOR, 1.98; 95% CI, 0.56-7.79).

**Conclusions and Relevance:**

The cardiometabolic comorbidity of psychosis and depression may have distinct, disorder-specific early-life origins. Disrupted insulin sensitivity could be a shared risk factor for comorbid cardiometabolic disorders and psychosis. A puberty-onset major increase in BMI could be a risk factor or risk indicator for adult depression. These markers may represent targets for prevention and treatment of cardiometabolic disorders in individuals with psychosis and depression.

## Key Points

**Question:**

Are longitudinal trends in insulin levels and body mass index from childhood associated with adult depression and psychosis?

**Findings:**

This cohort study of repeated-measure data from age 1 to 24 years in up to 10 463 individuals identified trajectories of fasting insulin levels and body mass index. Persistently high fasting insulin levels from age 9 years were associated with psychosis at 24 years, and puberty-onset body mass index increase was associated with depression at 24 years.

**Meaning:**

This study’s findings suggest that changes in insulin sensitivity and adiposity starting from childhood may have disorder-specific associations with psychosis and depression and represent targets for prevention and treatment of cardiometabolic disorders in people with psychosis and depression.

## Teaser

This cohort study examines changes in fasting insulin levels and body mass index from childhood to age 24 years and the risk for psychiatric disorders in that cohort at age 24 years.

## Introduction

Cardiometabolic disorders often occur concomitantly with depression and schizophrenia,^1^ leading to a reduced quality of life, increased health care costs,^2^ and a shortened life expectancy.^3,4^ Traditionally, this comorbidity has been attributed to chronic lifestyle factors (eg, physical inactivity or smoking) or the adverse effects of psychotropic medications.^5^ However, meta-analyses report altered glucose-insulin homeostasis in relatively young, drug-naive patients with first-episode psychosis.^6,7^ Similarly, reports from population-based longitudinal studies suggest a bidirectional association between depression and cardiovascular disease.^8,9^ Together, this evidence suggests that cardiometabolic and psychiatric conditions may share pathophysiologic mechanisms. However, 2 key issues remain.

First, existing studies have predominantly included prevalent depression or psychosis cases and so cannot appropriately test the direction of association between cardiometabolic and psychiatric phenotypes.^10^ Second, most longitudinal studies have included one-off measures of cardiometabolic indices, overlooking dynamic temporal changes in these markers.^11,12^ Longitudinal repeated measurements could provide a more reliable measure of underlying homeostatic mechanisms and could identify population subgroups. For example, aberrant trajectories of childhood body mass index (BMI) are associated with adult cardiometabolic disorders.^13^ Although cardiometabolic function encompasses a broad range of parameters, 2 pathways—insulin sensitivity and adiposity—are of particular interest regarding psychosis and depression. Genetic studies have indicated associations of BMI with depression^14^ and fasting insulin (FI) levels with schizophrenia.^15^ However, to our knowledge, no studies have examined whether FI level and BMI trajectories from childhood are associated with adult psychosis and depression.

Using data from the Avon Longitudinal Study of Parents and Children (ALSPAC) birth cohort, we aimed to (1) delineate longitudinal trajectories of FI level and BMI based on repeated measurements in individuals between ages 1 and 24 years, (2) examine the characteristics of identified trajectories, and (3) test associations with risks of psychosis and depression at 24 years in the total sample and by sex. We hypothesized that altered cardiometabolic development from childhood would be associated with increased risks for depression and psychosis in adulthood.

## Methods

### Cohort and Sample

The ALSPAC initially recruited 14 541 pregnant residents in southwest England, with expected delivery dates between April 1, 1991, and December 31, 1992, resulting in 14 062 live births.^16,17,18^ An additional 913 participants were recruited subsequently. Participants received financial compensation. Data were collected and managed using REDCap (University of Bristol^19,20^). Modeling of the trajectories was performed using 5790 participants for FI levels and 10 463 participants for BMI (eFigure 1 in the Supplement). Missing exposure data were handled using full-information maximum likelihood estimation (eMethods in the Supplement). Data were deidentified. The ALSPAC Ethics and Law Committee and local research ethics committees provided ethical approval for the ALSPAC cohort study. Ethical approval for the present study was obtained via the ALSPAC Executive Committee. Consent for biological samples was collected in accordance with the Human Tissue Act of 2004 covering England, Wales and Northern Ireland. Informed consent for all collected data was obtained from participants following the recommendations of the ALSPAC Ethics and Law Committee at the time. This study followed the Strengthening the Reporting of Observational Studies in Epidemiology (STROBE) reporting guideline for cohort study.

### Measurement of Exposures

Fasting insulin levels were measured at ages 9 (n = 894), 15 (n = 3484), 18 (n = 3286), and 24 (n = 3253) years, using an ultrasensitive automated microparticle enzyme immunoassay (Mercodia), which does not cross-react with proinsulin. Sensitivity of the immunoassay was 0.07 mU/L, and interassay and intraassay coefficients of variation were less than 6%. Fasting blood samples were drawn at 9 am after a 10-hour fast, then spun and stored at −80 °C. There was no evidence of freeze-thaw cycles during storage.

Body mass index, calculated as weight in kilograms divided by height in meters squared, was measured at 1 (n = 1236), 2 (n = 1036), 3 (n = 1050), 4 (n = 1018), 7 (n = 8200), 9 (n = 7633), 10 (n = 7465), 11 (n = 7100), 12 (n = 6704), 15 (n = 5415), 18 (n = 5061), and 24 (n = 3975) years.

### Psychiatric Outcomes at Age 24 Years

Psychotic experiences (PEs) were identified through the semistructured Psychosis-Like Symptom Interview^21^ conducted by trained psychology graduates and coded per the definitions in the Schedules for Clinical Assessment in Neuropsychiatry, version 2.0.^22^ The Psychosis-Like Symptom Interview had good interrater (intraclass correlation: 0.81; 95% CI, 0.68-0.89) and test-retest (0.9; 95% CI, 0.83-0.95) reliability. Psychotic experiences occurring in the past 6 months covered the 3 main positive symptom domains: hallucinations, delusions, and thought interference. After cross-questioning, interviewers rated PEs as absent, suspected, or definite. We included cases of definite PEs; the comparator group comprised individuals with suspected or absent PEs.

Cases of at-risk mental state were identified by mapping Psychosis-Like Symptom Interview data to Comprehensive Assessment of At-Risk Mental State (CAARMS) criteria.^23^ Cases were defined as participants meeting CAARMS criteria for attenuated psychosis (symptoms not reaching the psychosis threshold owing to levels of intensity or frequency) or brief limited intermittent psychosis (frank psychotic symptoms that resolved spontaneously within 1 week).

Cases of psychotic disorder were defined^21^ as definite PEs that were not attributable to sleep or fever, had occurred more than once per month during the previous 6 months, and were very distressing or negatively impactful on social/occupational functioning, and led to seeking of professional help. We also included participants meeting the criteria for CAARMS psychotic disorder (threshold psychotic symptoms occurring for >1 week).

Ten questions from the Community Assessment of Psychic Experiences questionnaire^24^ were administered covering interest, motivation, emotional reactivity, pleasure, and sociability. Participants rated each item as 0 (never), 1 (sometimes), 2 (often), and 3 (always). We recoded the variables by scoring always and often as 1 and never and sometimes as 0, and then summed the values to result in a possible total score of 0 to 10.

Depression was measured using the computerized Clinical Interview Schedule–Revised.^25^ The interview assesses symptoms of depression occurring in the past week and provides a diagnosis of depressive episode based on the *International Statistical Classification of Diseases, Tenth Revision* criteria, which we used as a binary outcome (codes F32.0-32.2). We also included a Clinical Interview Schedule–Revised depression severity score, comprising scores for mood, thoughts, fatigue, concentration, and sleep, as a continuous outcome.

For assessment of potential confounders, we included sex at birth, race/ethnicity, paternal social class, childhood emotional and behavioral problems (measured using the Strength and Difficulties Questionnaire^26^ at age 7 years), and cumulative scores of smoking, physical activity, alcohol use, substance use, sleep problems, and average calorie intake between ages 7 and 24 years (eMethods in the Supplement).

### Statistical Analysis

We standardized (*z* transformed) FI levels and BMI separately in males and females and then combined the sex-stratified *z* scores for each variable at each time point to delineate trajectories using curvilinear growth mixture modeling^27^ (eMethods in the Supplement). We used *z* scores to measure the relative change in FI levels and BMI because BMI increases in all young people during early life. Because the sample size for FI levels at age 9 years was smaller, we repeated growth mixture modeling without age-9-years data and compared the characteristics of the resultant trajectories. Analyses were conducted using MPlus, version 8 (Muthén & Muthén), and R, version 3.6.0 (R Project for Statistical Computing). Two-tailed *P* values were corrected for multiple testing using the Holm-Bonferroni method^28^ for the 6 psychiatric outcomes. A corrected *P* value <.05 was used as the threshold for significance. We estimated how participants overlapped between BMI and FI level trajectories (the most common and highest risk) using the φ correlation coefficient.

We used the 3-step method^29^ to estimate associations of sociodemographic, lifestyle, and clinical factors with trajectory membership (eMethods in the Supplement). The 3-step method allows class separation unaffected by auxiliary variables, retains and includes information on class uncertainty, and is robust when entropy is greater than 0.60. Multinomial regression was used to estimate odds ratios (ORs) and 95% CIs for the associations of sociodemographic and lifestyle factors with FI level and BMI trajectories compared with the most common trajectory. We considered time-invariant (sex, ethnicity, social class at birth, family history of cardiovascular disease, gestational age, birth weight, and perinatal stressful life events) and time-variant (physical activity and smoking in adolescence and early adulthood) factors. Odds ratios represent the increase in the risk of membership of a particular trajectory category per SD increase in factor. Next, we examined the clinical phenotype of trajectories at 24 years, examining mean levels of commonly measured clinical and biochemical factors for participants grouped by most-likely trajectory membership (eMethods in the Supplement). Next, we used logistic regression to estimate the association of trajectory membership with an age-appropriate cardiometabolic outcome: metabolic syndrome at 24 years (eMethods in the Supplement).

Using the 3-step method, logistic regression was used to estimate ORs and 95% CIs for binary outcomes per trajectory, compared with the most common trajectory. Linear regression for continuous outcomes was used to estimate β coefficients and 95% CIs representing the SD increase in the risk of outcomes per trajectory. We tested associations for the total sample and separately by sex before and after adjusting for potential confounders. Regression models for negative symptoms were additionally adjusted for depressive symptoms, and vice versa.

## Results

### Trajectories of FI Levels and BMI

**Figure 1.**
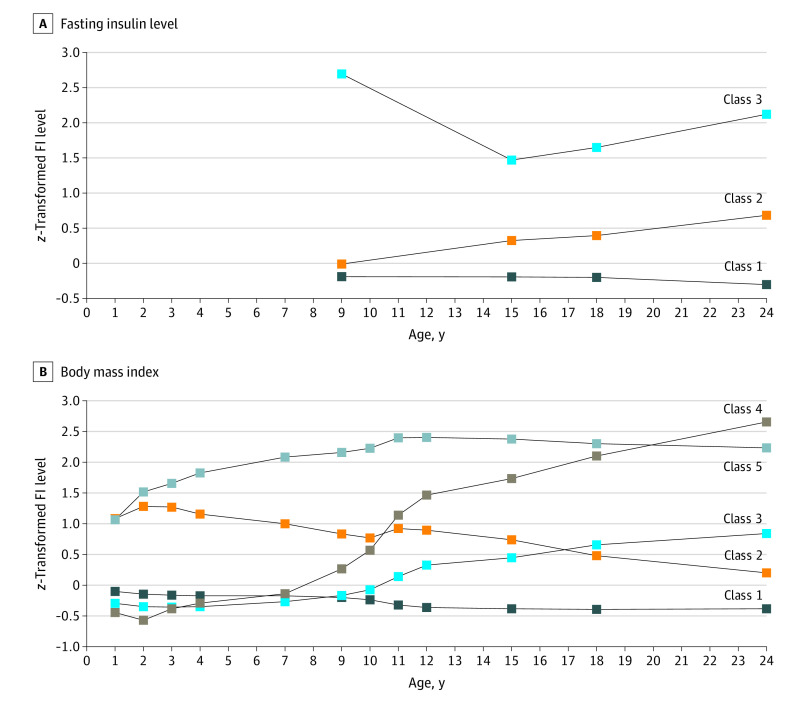
Fasting Insulin Levels and Body Mass Index Trajectories in the Avon Longitudinal Study of Parents and Children A, Fasting insulin levels measured at ages 9 to 24 years in 5790 participants. Class 1 (stable average) comprised 77.8% of the sample; class 2 (minor increase), 19.0%; and class 3 (persistently high), 3.1%. B, Body mass index measured at ages 1 to 24 years in 10 463 participants. Class 1 (stable average) comprised 71.1% of the sample; class 2 (gradually decreasing), 7.0%; class 3 (puberty-onset minor increase), 14.5%; class 4 (puberty-onset major increase), 1.9%; and class 5 (persistently high), 5.5%. Trajectories were delineated using growth mixture modeling at 4 time points for fasting insulin and 12 time points for body mass index. Nodes in the graph represent mean *z* scores for fasting insulin level or body mass index at each time point for each developmental trajectory.

Based on 5790 participants (2658 [45.9%] male, 3132 [54.1%] female), the 3-trajectory solution for FI levels was optimum, representing stable average (class 1: 4939 [77.8%]), minor increase (class 2: 693 [19.0%]), and persistently high (class 3: 158 [3.1%]) trajectories between ages 9 and 24 years (Figure 1A; eTable 1 and eFigure 2 in the Supplement). The trajectories were similar after excluding age-9-years data (eFigure 3 in the Supplement).

Based on 10 463 participants (5336 [51.0%] female, 5127 [49.0%] male) included in the analysis of BMI, the 5-trajectory solution was optimum, representing stable average (class 1: 8383 [71.1%]), gradually decreasing (class 2: 949 [7.0%]), puberty-onset minor increase (class 3: 668 [14.5%]), puberty-onset major increase (class 4: 174 [1.9%]), and persistently high (class 5: 289 [5.5%]) BMI trajectories between ages 1 and 24 years (Figure 1B; eTable 2 and eFigure 4 in the Supplement).

The stable average FI level and BMI trajectories were statistically significantly correlated (*r*_φ_ = 0.233, *P* < .001), as were the persistently high trajectories (*r*_φ_ = 0.092, *P* < .001).

Both deviating FI level trajectories were associated with lower social class, family history of cardiometabolic disease, lower physical activity, and smoking in adolescence and early adulthood. Lower birth weight and more perinatal stressful life events were associated with the persistently high trajectory compared with the stable average trajectory (eTable 3 in the Supplement). The persistently high trajectory cohort also had mean FI, high-density lipoprotein cholesterol, triglyceride, and C-reactive protein levels outside of UK reference ranges at 24 years (eTable 4 in the Supplement). Deviating FI level trajectories were associated with metabolic syndrome at 24 years (adjusted OR [aOR] for the persistently high trajectory, 9.21; 95% CI, 3.77-20.15) (eTable 5 in the Supplement).

Deviating BMI trajectories were associated with lower social class, family history of cardiometabolic disease, more perinatal stressful life events, lower physical activity, and smoking in adolescence and early adulthood compared with the stable average trajectory. Higher birth weight was associated with the gradually decreasing and persistently high trajectories, whereas lower birth weight was weakly associated with both puberty-onset increase trajectories (eTable 6 in the Supplement). Deviating BMI trajectories were also associated with mean values of waist circumference and FI, high-density lipoprotein cholesterol, and C-reactive protein levels outside of UK reference ranges at 24 years (eTable 7 in the Supplement). All deviating BMI trajectories were associated with metabolic syndrome at 24 years (aOR for the persistently high trajectory, 10.62; 95% CI, 5.89-19.13) (eTable 5 in the Supplement).

### Associations of FI and BMI Trajectories With Psychiatric Outcomes 

**Figure 2.**
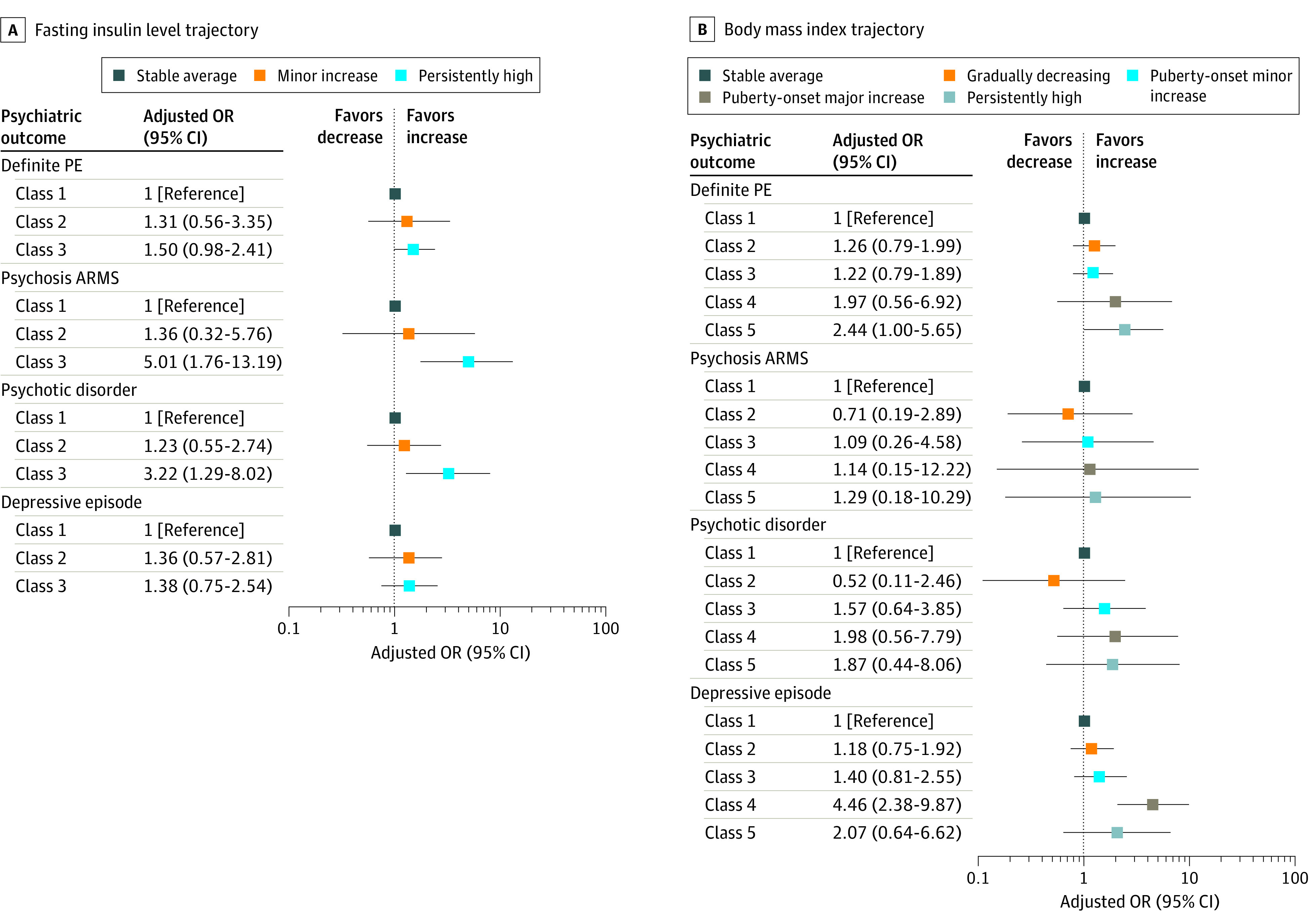
Associations of Fasting Insulin Levels and Body Mass Index Trajectories With Psychosis and Depressive Outcomes in the Avon Longitudinal Study of Parents and Children Adjusted odds ratios (ORs) and 95% CIs showing associations of fasting insulin (A) and body mass index (B) trajectories from childhood with risk of psychosis and depression outcomes at age 24 years after adjusting for sex, ethnicity, social class, childhood emotional and behavioral problems, and cumulative scores of smoking, physical activity, alcohol and substance use, sleep problems, and calorie intake. ARMS indicates at-risk mental state; PE, psychotic experiences.

The persistently high FI level trajectory was associated with the psychosis at-risk mental state (aOR, 5.01; 95% CI, 1.76-13.19), psychotic disorder (aOR, 3.22; 95% CI, 1.29-8.02), and negative symptoms (adjusted β, 0.07; 95% CI, 0.01-0.13) at age 24 years. Fasting insulin level trajectories were not associated with depression (aOR, 1.38; 95% CI, 0.75-2.54) (Table 1; Figure 2A; eTable 8 in the Supplement).

**Table 1.** Psychosis and Depressive Outcomes at Age 24 Years Associated With Fasting Insulin Level Trajectories From Age 9 to 24 Years

Trajectory and outcome at 24 y	Sample, No.	Odds ratio (95% CI)		*P* value^b^
		Unadjusted	Adjusted^a^	
**Definite PE**				
Class 1: stable average	4939	1 [Reference]	1 [Reference]	
Class 2: minor increase	693	1.48 (0.98-2.24)	1.31 (0.56-3.35)	>.99
Class 3: persistently high	158	1.88 (1.05-3.60)	1.50 (0.98-2.41)	.33
**Psychosis at-risk mental state **				
Class 1: stable average	4939	1 [Reference]	1 [Reference]	
Class 2: minor increase	693	1.59 (0.20-8.02)	1.36 (0.32-5.76)	>.99
Class 3: persistently high	158	6.33 (1.97-20.30)	5.01 (1.76-13.19)	.006
**Psychotic disorder **				
Class 1: stable average	4939	1.00 [reference]	1.00 [Reference]	
Class 2: minor increase	693	1.85 (0.70-4.88)	1.23 (0.55-2.74)	>.99
Class 3: persistently high	158	4.74 (1.67-13.42)	3.22 (1.29-8.02)	.05
**Depressive episode**				
Class 1: stable average	4939	1 [Reference]	1 [Reference]	
Class 2: minor increase	693	1.26 (0.73-2.67)	1.36 (0.57-2.81)	.88
Class 3: persistently high	158	1.31 (0.81-4.32)	1.38 (0.75-2.54)	.69

Abbreviation: PE, psychotic experience.

^a^ Adjusted for sex, ethnicity, social class, Strength and Difficulties Questionnaire (measured at 7 years) findings, and cumulative scores for smoking, physical activity, alcohol and substance use, sleep problems, and calorie intake.

^b^ *P* values adjusted for multiple testing using the Holm-Bonferroni method.

The puberty-onset major increase trajectory of BMI was associated with a higher risk of a depressive episode (aOR, 4.46; 95% CI, 2.38-9.87) and depressive symptoms (adjusted β, 0.08; 95% CI, 0.03-0.14) at age 24 years. The puberty-onset minor increase trajectory was weakly associated with depressive symptoms at 24 years (adjusted β, 0.06; 95% CI, 0.01-0.11). Body mass index trajectories were not associated with psychosis outcomes (aOR for psychotic disorder in the puberty-onset major increase trajectory, 1.98; 95% CI, 0.56-7.79) (Table 2 and Figure 2B; eTable 9 in the Supplement).

**Table 2.** Psychiatric Outcomes at Age 24 Years Associated With BMI Trajectories From Age 1 to 24 Years

Trajectory and outcome at 24 y	Sample, No.	Odds ratio (95% CI)		*P* value^b^
		Unadjusted	Adjusted^a^	
**Definite PE**				
Class 1: stable average	8383	1 [Reference]	1 [Reference]	
Class 2: gradually decreasing	949	1.43 (0.82-1.96)	1.26 (0.79-1.99)	>.99
Class 3: puberty-onset minor increase	668	1.66 (0.87-2.55)	1.22 (0.79-1.89)	>.99
Class 4: puberty-onset major increase	174	3.56 (0.87-11.54)	1.97 (0.56-6.92)	>.99
Class 5: persistently high	289	3.21 (1.01-9.11)	2.44 (1.00-5.65)	.37
**Psychosis at-risk mental state**				
Class 1: stable average	8383	1 [Reference]	1 [Reference]	
Class 2: gradually decreasing	949	0.49 (0.10-3.21)	0.71 (0.19-2.89)	>.99
Class 3: puberty-onset minor increase	668	1.12 (0.23-5.43)	1.09 (0.26-4.58)	>.99
Class 4: puberty-onset major increase	174	1.32 (0.10-13.11)	1.14 (0.15-12.22)	>.99
Class 5: persistently high	289	1.55 (0.44-3.21)	1.29 (0.18-10.29)	>.99
**Psychotic disorder **				
Class 1: stable average	8383	1 [Reference]	1 [Reference]	
Class 2: gradually decreasing	949	0.44 (0.21-2.03)	0.52 (0.11-2.46)	>.99
Class 3: puberty-onset minor increase	668	1.97 (0.60-3.46)	1.57 (0.64-3.85)	>.99
Class 4: puberty-onset major increase	174	2.14 (0.65-6.21)	1.98 (0.56-7.79)	>.99
Class 5: persistently high	289	3.11 (0.53-13.22)	1.87 (0.44-8.06)	>.99
**Depressive episode **				
Class 1: stable average	8383	1 [Reference]	1 [Reference]	
Class 2: gradually decreasing	949	1.33 (0.77-1.88)	1.18 (0.75-1.92)	>.99
Class 3: puberty-onset minor increase	668	1.69 (0.90-3.21)	1.40 (0.81-2.55)	>.99
Class 4: puberty-onset major increase	174	8.91 (4.21-17.12)	4.46 (2.38-9.87)	.006
Class 5: persistently high	289	3.01 (0.91-7.59)	2.07 (0.64-6.62)	>.99

Abbreviations: BMI, body mass index; PE, psychotic experience.

^a^ Adjusted for sex, ethnicity, social class, Strength and Difficulties Questionnaire (measured at 7 years), and cumulative scores for smoking, physical activity, alcohol and substance use, sleep problems, and calorie intake.

^b^ *P* values adjusted for multiple testing using the Holm-Bonferroni method.

### Sex-Stratified Associations of Risks for Psychiatric Outcomes

**Figure 3.**
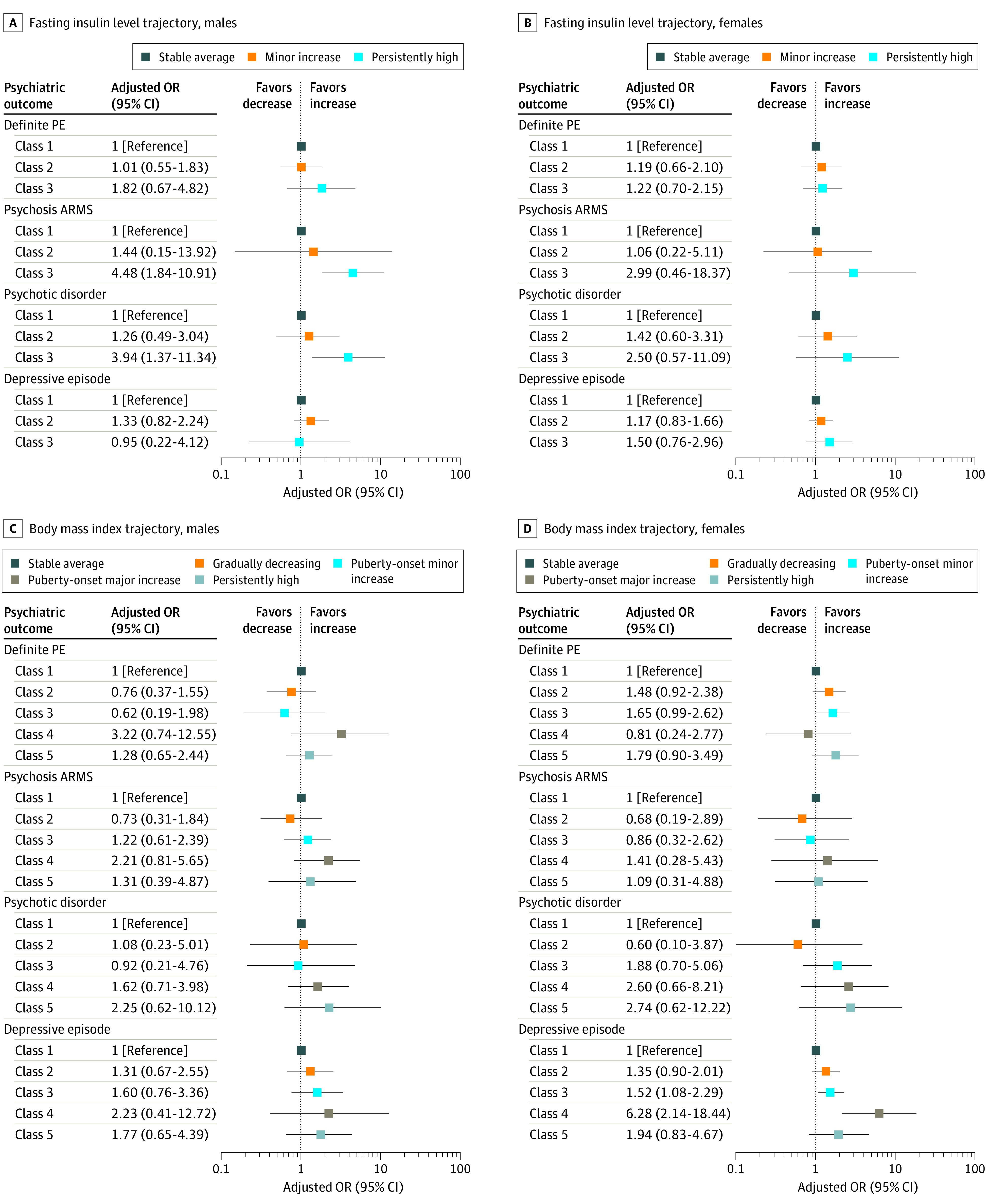
Sex-Stratified Associations of Fasting Insulin Levels and Body Mass Index Trajectories With Psychosis and Depressive Outcomes in the Avon Longitudinal Study of Parents and Children Adjusted odds ratios (ORs) and 95% CIs showing associations of fasting insulin level trajectories in males (A) and females (B) and body mass index trajectories in males (C) and females (D) from childhood with risk of psychosis and depression outcomes at age 24 years after adjusting for sex, ethnicity, social class, childhood emotional and behavioral problems, and cumulative scores of smoking, physical activity, alcohol and substance use, sleep problems, and calorie intake. ARMS indicates at-risk mental state; PE, psychotic experience.

For FI trajectories, the pattern of association with risks for psychiatric outcomes in sex-stratified analysis was similar to the primary analysis. For example, point estimates for the association between the persistently high FI trajectory and psychotic disorder were similar in males (aOR, 3.94; 95% CI, 1.10-11.96) compared with females (aOR, 2.50; 95% CI, 0.57-11.09), and 95% CIs overlapped. There was no association between persistently high FI and depression in males (aOR, 0.95; 95% CI, 0.22-4.12) or females (aOR, 1.50; 95% CI, 0.76-2.96) (Figure 3; eTable 10 and eTable 11 in the Supplement). For BMI, point estimates for depression for both puberty-onset increase trajectories were larger in females. For example, for the puberty-onset major increase trajectory, the association for females (aOR, 6.28; 95% CI, 2.14-18.44) was stronger than for males (aOR, 2.23; 95% CI, 0.41-12.72). There was no significant association of BMI trajectories with psychosis outcomes. For example, there was no association between puberty-onset major BMI increase and psychotic disorder for males (aOR, 1.62; 95% CI, 0.71-3.98) or females (aOR, 2.60; 95% CI, 0.66-8.21) (Figure 3; eTable 12 and eTable 13 in the Supplement).

## Discussion

We delineated FI level and BMI trajectories from early life, using prospective repeated measurements in a large population-representative birth cohort, and report distinct associations with psychosis and depression measured in adulthood. After adjusting for a number of relevant confounders, we found that persistently high FI levels from mid-childhood appeared to be associated with an increased risk of psychosis outcomes at age 24 years, while BMI increases around the age of puberty onset were associated with depression at age 24 years. Associations of BMI and FI level trajectories with cardiometabolic risk factors, such as social class, ethnicity, smoking, physical activity, and adult metabolic syndrome, suggest face validity to the identified trajectories. Although the last data point for BMI and FI levels overlapped with the outcome assessment, the trajectories were differentiated by mid-childhood, suggesting a temporal association between exposure and outcome. Evidence for the association of puberty-onset BMI increase and adult depression remained after adjusting for childhood emotional and behavioral problems, suggesting that a reverse direction of association may not fully explain this finding. Although the same adjustment may be less capable of ruling out reverse direction of the association between persistently high FI levels and psychosis, it is unlikely that many participants had experienced psychosis before age 9 years, and so a reverse direction of association is unlikely.

We found consistent evidence for an association between FI level trajectories and psychosis outcomes. Effect sizes were largest in the persistently high trajectory, consistent with a dose-response relationship, and point estimates were larger in more clinically relevant outcomes. Our findings complement meta-analyses reporting altered glucose-insulin homeostasis in first-episode psychosis.^6,7^ Moreover, our results suggest that disruptions to glucose-insulin homeostasis detectable at first-episode psychosis in adults may begin in childhood. The point estimates partly attenuated after adjustment for confounders, suggesting that malleable lifestyle factors, such as smoking, physical activity, and diet, should remain targets for reducing the risk of incident cardiometabolic disorders in young people with psychosis. We also found that participants classified into the persistently high FI level trajectory, who had the highest risk of psychosis, had mean BMI and fasting plasma glucose values within reference ranges at age 24 years. Therefore, the risk of incident cardiometabolic disorders in these individuals may not be detected in psychosis early-intervention services, since commonly measured physical indices may not identify them. Consequently, careful assessment and clinical considerations are needed to minimize the risk of cardiometabolic disorders in these individuals.

Our findings suggest that altered glucose-insulin homeostasis could be a shared mechanism for psychosis and type 2 diabetes, which could be genetic and/or environmental in origin. People with comorbid schizophrenia and type 2 diabetes have a higher genetic predisposition for both disorders compared with controls,^30^ and genetic predisposition for schizophrenia is associated with insulin resistance in patients with schizophrenia.^31^ In addition, we found that the persistently high FI trajectory, which had the strongest associations with psychosis outcomes, was also associated with lower birth weight and perinatal stressful life events. We noted similar patterns of association in BMI trajectories that were associated with depression. These findings are consistent with the fetal programming hypothesis,^32^ which posits that disruption in early-life development can have broad influences on adult health.

Our findings regarding the association of BMI trajectories with depression at age 24 years are in line with meta-analyses^33,34^ suggesting an association between BMI and risk of depression. Similar trajectories of BMI have been linked with adult type 2 diabetes,^35^ obesity,^36^ and coronary heart disease.^37^ The character and composition of BMI trajectories we identified are consistent with those of previous studies, although our length of follow-up was longer than the follow-up of most previous studies.^38^

Our findings provide further insights into the link between BMI and depression,^34^ suggesting that puberty-onset increases in BMI specifically are associated with risk of adult depression. This finding, together with the lack of evidence for an association between persistently high BMI and depression, indicates that BMI might be a risk indicator for depression rather than a risk factor because individuals in the persistently high BMI trajectory would likely have been exposed to the “largest dose” of BMI. Therefore, if BMI were the risk factor, one would have expected the largest effect size for depression in that trajectory. Consequently, environmental and/or genetic factors influencing BMI during puberty are likely to be important risk factors for depression. For instance, social stressors, such as bullying, may predispose to altered eating behaviors and an increased risk of depression in adolescents.^39^ In addition, deviating childhood BMI trajectories have been associated with a greater risk of adolescent and adult eating disorders,^40^ which are commonly comorbid with depression.^41^ Also, the effects of estrogen may be relevant, since the associations of puberty-onset BMI increases and depression appeared to be stronger in females than males. Changes in estrogen levels are associated with depressive symptoms throughout life in women, including pregnancy,^42^ menopause,^43^ and puberty.^44^ Estrogen is associated with obesity^45^ and may explain the genetic correlation of age at menarche with adult obesity^46^ and depression.^47^ Further research is needed to identify factors influencing pubertal BMI increases, as they may represent important preventive targets for depression.

We did not find consistent evidence for associations of FI level trajectories with depression or of BMI trajectories with psychosis. Previous research has reported mixed findings regarding the association between insulin resistance and depression in young adults.^48,49^ However, some estimates for the associations of BMI trajectories with psychosis outcomes in our analyses had wide 95% CIs, possibly owing to sample size. These particular findings require replication in larger samples of people with psychosis.

### Strengths and Limitations

Strengths of the study include a longitudinal design with repeated measurements of BMI and FI levels between ages 1 and 24 years in a relatively large sample enabling a detailed examination of dynamic cardiometabolic changes from childhood to early adulthood. We included several relevant depression and psychosis outcomes, which allowed us to examine for specificity and for a biological gradient of evidence.

Limitations of the study include missing data. Although we used a robust method to handle missing data, full-information maximum likelihood may be biased in instances in which data were not missing at random.^50^ However, the risk of bias in full-information maximum likelihood is no greater than the bias associated with traditional complete-case methods,^51^ and full-information maximum likelihood permitted a larger sample size and therefore increased statistical power. Nevertheless, missing psychiatric outcome data may have affected our results. Furthermore, although we adjusted for a number of relevant potential confounders, residual confounding could still be an issue. For example, we could not account for psychological stress since data on cortisol levels were available only at age 9 years in a small subsection of the cohort. Therefore, further research is needed, such as mendelian randomization analysis, to examine for potentially unconfounded associations. In addition, the 95% CIs were relatively wide for the sex-stratified analysis, likely owing to reduced statistical power. Therefore, replication of our work in larger samples is required. In addition, the ALSPAC data set does not include *International Statistical Classification of Diseases* and *DSM* diagnoses of schizophrenia. However, our psychotic disorder outcome would likely meet the threshold for clinical intervention, and all our psychosis outcomes lie on the schizophrenia continuum.

## Conclusions

We report that the cardiometabolic comorbidity of psychosis and depression may have distinct early-life origins. Disrupted insulin sensitivity from mid-childhood appeared to be associated with adult psychosis, and BMI increases starting around the time of puberty onset were associated with adult depression. Although residual confounding may be an issue, our results suggest that these cardiometabolic markers could be among shared risk factors and indicators for adult cardiometabolic and psychiatric disorders and may represent novel targets for prevention and treatment of cardiometabolic disorders in people with psychosis and depression.

## Notes

Supplement. **eMethods.** Detailed Methods **eTable 1.** Growth Mixture Model Fit Indices for Fasting Insulin Level **eTable 2.** Growth Mixture Model Fit Indices for Body Mass Index **eTable 3.** Odds Ratios for Multinomial Logistic Regression Analyses Examining Predictors of Membership of Fasting Insulin Trajectories **eTable 4.** Anthropometric and Biochemical Characteristics at Age 24 of Participants included in Fasting Insulin Trajectory Classes **eTable 5.** Odds Ratios for Metabolic Syndrome at Age 24 Associated with Fasting Insulin and Body Mass Index Trajectories **eTable 6.** Odds Ratios for Multinomial Regression Analyses Examining Predictors of Membership of Body Mass Index Trajectories **eTable 7.** Anthropometric and Biochemical Characteristics at Age 24 of Participants included in Body Mass Index Trajectory Classes **eTable 8.** Odds Ratios for Associations of Fasting Insulin Trajectories with Continuous Psychiatric Outcomes **eTable 9.** Odds Ratios for Associations of Body Mass Index Trajectories with Continuous Psychiatric Outcomes **eTable 10.** Odds Ratios for Sex-Stratified Associations of Fasting Insulin Trajectories with Binary Psychiatric Outcomes **eTable 11.** Odds Ratios for Sex-Stratified Associations of Fasting Insulin Trajectories with Continuous Psychiatric Outcomes **eTable 12.** Odds Ratios for Sex-Stratified Associations of Body Mass Index Trajectories with Binary Psychiatric Outcomes **eTable 13.** Odds Ratios for Sex-Stratified Associations of Body Mass Index Trajectories with Continuous Psychiatric Outcomes **eFigure 1**. Flowchart of Available Sample for Primary Analyses **eFigure 2.** Trajectory Means and Individual Values per Developmental Trajectory of Fasting Insulin **eFigure 3.** Sensitivity Analysis Examining Trajectories of Fasting Insulin Between Ages 15-24 **eFigure 4.** Trajectory Means and Individual Values per Developmental Trajectory of Body Mass Index **eReferences**

## References

[1] Firth J, Siddiqi N, Koyanagi A, . *The Lancet Psychiatry* Commission: a blueprint for protecting physical health in people with mental illness. Lancet Psychiatry. 2019;6(8):675-712. doi:10.1016/S2215-0366(19)30132-4 31324560

[2] Naylor C, Parsonage M, McDaid D, Knapp M, Fossey M, Galea A. Long-term Conditions and Mental Health: the Cost of Co-morbidities. The King’s Fund; 2012.

[3] Laursen TM, Plana-Ripoll O, Andersen PK, . Cause-specific life years lost among persons diagnosed with schizophrenia: is it getting better or worse? Schizophr Res. 2019;206:284-290. doi:10.1016/j.schres.2018.11.003 30446270

[4] Plana-Ripoll O, Pedersen CB, Agerbo E, . A comprehensive analysis of mortality-related health metrics associated with mental disorders: a nationwide, register-based cohort study. Lancet. 2019;394(10211):1827-1835. doi:10.1016/S0140-6736(19)32316-5 31668728

[5] Leucht S, Cipriani A, Spineli L, . Comparative efficacy and tolerability of 15 antipsychotic drugs in schizophrenia: a multiple-treatments meta-analysis. Lancet. 2013;382(9896):951-962. doi:10.1016/S0140-6736(13)60733-3 23810019

[6] Perry BI, McIntosh G, Weich S, Singh S, Rees K. The association between first-episode psychosis and abnormal glycaemic control: systematic review and meta-analysis. Lancet Psychiatry. 2016;3(11):1049-1058. doi:10.1016/S2215-0366(16)30262-0 27720402

[7] Pillinger T, Beck K, Gobjila C, Donocik JG, Jauhar S, Howes OD. Impaired glucose homeostasis in first-episode schizophrenia: a systematic review and meta-analysis. JAMA Psychiatry. 2017;74(3):261-269. doi:10.1001/jamapsychiatry.2016.3803 28097367 PMC6352957

[8] Penninx BW, Beekman AT, Honig A, . Depression and cardiac mortality: results from a community-based longitudinal study. Arch Gen Psychiatry. 2001;58(3):221-227. doi:10.1001/archpsyc.58.3.221 11231827

[9] van Melle JP, de Jonge P, Spijkerman TA, . Prognostic association of depression following myocardial infarction with mortality and cardiovascular events: a meta-analysis. Psychosom Med. 2004;66(6):814-822. doi:10.1097/01.psy.0000146294.82810.9c 15564344

[10] Kucukgoncu S, Kosir U, Zhou E, Sullivan E, Srihari VH, Tek C. Glucose metabolism dysregulation at the onset of mental illness is not limited to first episode psychosis: A systematic review and meta-analysis. Early Interv Psychiatry. 2019;13(5):1021-1031. doi:10.1111/eip.12749 30277314 PMC6445792

[11] Perry BI, Upthegrove R, Thompson A, . Dysglycaemia, inflammation and psychosis: findings from the UK ALSPAC Birth Cohort. Schizophr Bull. 2019;45(2):330-338. doi:10.1093/schbul/sby040 29635418 PMC6403055

[12] Mannan M, Mamun A, Doi S, Clavarino A. Prospective associations between depression and obesity for adolescent males and females—a systematic review and meta-analysis of longitudinal studies. PLoS One. 2016;11(6):e0157240. doi:10.1371/journal.pone.0157240 27285386 PMC4902254

[13] Buscot MJ, Thomson RJ, Juonala M, . Distinct child-to-adult body mass index trajectories are associated with different levels of adult cardiometabolic risk. Eur Heart J. 2018;39(24):2263-2270. doi:10.1093/eurheartj/ehy161 29635282

[14] Tyrrell J, Mulugeta A, Wood AR, . Using genetics to understand the causal influence of higher BMI on depression. Int J Epidemiol. 2019;48(3):834-848. doi:10.1093/ije/dyy223 30423117 PMC6659462

[15] Li Z, Chen P, Chen J, . Glucose and insulin-related traits, type 2 diabetes and risk of schizophrenia: a mendelian randomization study. EBioMedicine. 2018;34:182-188. doi:10.1016/j.ebiom.2018.07.037 30100396 PMC6116472

[16] Boyd A, Golding J, Macleod J, . Cohort profile: the “children of the 90s”—the index offspring of the Avon Longitudinal Study of Parents and Children. Int J Epidemiol. 2013;42(1):111-127. doi:10.1093/ije/dys064 22507743 PMC3600618

[17] Fraser A, Macdonald-Wallis C, Tilling K, . Cohort profile: the Avon Longitudinal Study of Parents and Children: ALSPAC mothers cohort. Int J Epidemiol. 2013;42(1):97-110. doi:10.1093/ije/dys066 22507742 PMC3600619

[18] Northstone K, Lewcock M, Groom A, . The Avon Longitudinal Study of Parents and Children (ALSPAC): an update on the enrolled sample of index children in 2019. Wellcome Open Res. 2019;4:51. doi:10.12688/wellcomeopenres.15132.1 31020050 PMC6464058

[19] Harris PA, Taylor R, Minor BL, ; REDCap Consortium. The REDCap consortium: building an international community of software platform partners. J Biomed Inform. 2019;95:103208. doi:10.1016/j.jbi.2019.103208 31078660 PMC7254481

[20] Harris PA, Taylor R, Thielke R, Payne J, Gonzalez N, Conde JG. Research electronic data capture (REDCap)—a metadata-driven methodology and workflow process for providing translational research informatics support. J Biomed Inform. 2009;42(2):377-381. doi:10.1016/j.jbi.2008.08.010 18929686 PMC2700030

[21] Sullivan SA, Kounali D, Cannon M, . A Population-based cohort study examining the incidence and impact of psychotic experiences from childhood to adulthood, and prediction of psychotic disorder. Am J Psychiatry. 2020;177(4):308-317. doi:10.1176/appi.ajp.2019.19060654 31906710

[22] World Health Organization. Division of Mental Health. Schedules for clinical assessment in neuropsychiatry: version 2. World Health Organization. Published 1994. Accessed December 7, 2020. https://apps.who.int/iris/handle/10665/40356

[23] Yung AR, Yuen HP, McGorry PD, . Mapping the onset of psychosis: the Comprehensive Assessment of At-Risk Mental States. Aust N Z J Psychiatry. 2005;39(11-12):964-971. doi:10.1080/j.1440-1614.2005.01714.x 16343296

[24] Konings M, Bak M, Hanssen M, van Os J, Krabbendam L. Validity and reliability of the CAPE: a self-report instrument for the measurement of psychotic experiences in the general population. Acta Psychiatr Scand. 2006;114(1):55-61. doi:10.1111/j.1600-0447.2005.00741.x 16774662

[25] Lewis G, Pelosi AJ, Araya R, Dunn G. Measuring psychiatric disorder in the community: a standardized assessment for use by lay interviewers. Psychol Med. 1992;22(2):465-486. doi:10.1017/S0033291700030415 1615114

[26] Goodman R. Psychometric properties of the strengths and difficulties questionnaire. J Am Acad Child Adolesc Psychiatry. 2001;40(11):1337-1345. doi:10.1097/00004583-200111000-00015 11699809

[27] Ram N, Grimm KJ. Growth mixture modeling: a method for identifying differences in longitudinal change among unobserved groups. Int J Behav Dev. 2009;33(6):565-576. doi:10.1177/0165025409343765 23885133 PMC3718544

[28] Holm S. A simple sequentially rejective multiple test procedure. Scand J Stat. 1979;6(2):65-70.

[29] Asparouhov T, Muthén B. Auxiliary variables in mixture modeling: three-step approaches using Mplus. Struct Equ Modeling. 2014;21(3):329-341. doi:10.1080/10705511.2014.915181

[30] Hackinger S, Prins B, Mamakou V, . Evidence for genetic contribution to the increased risk of type 2 diabetes in schizophrenia. Transl Psychiatry. 2018;8(1):252. doi:10.1038/s41398-018-0304-6 30470734 PMC6251918

[31] Tomasik J, Lago SG, Vázquez-Bourgon J, . Association of insulin resistance with schizophrenia polygenic risk score and response to antipsychotic treatment. JAMA Psychiatry. 2019;76(8):864-867. doi:10.1001/jamapsychiatry.2019.0304 30942838 PMC6583823

[32] Barker DJ, Gluckman PD, Godfrey KM, Harding JE, Owens JA, Robinson JS. Fetal nutrition and cardiovascular disease in adult life. Lancet. 1993;341(8850):938-941. doi:10.1016/0140-6736(93)91224-A 8096277

[33] Gariepy G, Nitka D, Schmitz N. The association between obesity and anxiety disorders in the population: a systematic review and meta-analysis. Int J Obes (Lond). 2010;34(3):407-419. doi:10.1038/ijo.2009.252 19997072

[34] Luppino FS, de Wit LM, Bouvy PF, . Overweight, obesity, and depression: a systematic review and meta-analysis of longitudinal studies. Arch Gen Psychiatry. 2010;67(3):220-229. doi:10.1001/archgenpsychiatry.2010.2 20194822

[35] Zhang T, Xu J, Li S, . Trajectories of childhood BMI and adult diabetes: the Bogalusa Heart Study. Diabetologia. 2019;62(1):70-77. doi:10.1007/s00125-018-4753-5 30343393 PMC6365010

[36] Rolland-Cachera MF, Péneau S. Growth trajectories associated with adult obesity. World Rev Nutr Diet. 2013;106:127-134. 23428691 10.1159/000342564

[37] Barker DJ, Osmond C, Forsén TJ, Kajantie E, Eriksson JG. Trajectories of growth among children who have coronary events as adults. N Engl J Med. 2005;353(17):1802-1809. doi:10.1056/NEJMoa044160 16251536

[38] Mattsson M, Maher GM, Boland F, Fitzgerald AP, Murray DM, Biesma R. Group-based trajectory modelling for BMI trajectories in childhood: a systematic review. Obes Rev. 2019;20(7):998-1015. doi:10.1111/obr.12842 30942535

[39] Lee KS, Vaillancourt T. Longitudinal associations among bullying by peers, disordered eating behavior, and symptoms of depression during adolescence. JAMA Psychiatry. 2018;75(6):605-612. doi:10.1001/jamapsychiatry.2018.0284 29641816 PMC6137525

[40] Yilmaz Z, Gottfredson NC, Zerwas SC, Bulik CM, Micali N. Developmental premorbid body mass index trajectories of adolescents with eating disorders in a longitudinal population cohort. J Am Acad Child Adolesc Psychiatry. 2019;58(2):191-199. doi:10.1016/j.jaac.2018.11.008 30738546 PMC6766404

[41] Welch E, Jangmo A, Thornton LM, . Treatment-seeking patients with binge-eating disorder in the Swedish national registers: clinical course and psychiatric comorbidity. BMC Psychiatry. 2016;16:163. doi:10.1186/s12888-016-0840-7 27230675 PMC4880842

[42] Schiller CE, Meltzer-Brody S, Rubinow DR. The role of reproductive hormones in postpartum depression. CNS Spectr. 2015;20(1):48-59. doi:10.1017/S1092852914000480 25263255 PMC4363269

[43] Dalal PK, Agarwal M. Postmenopausal syndrome. Indian J Psychiatry. 2015;57(suppl 2):S222-S232. doi:10.4103/0019-5545.161483 26330639 PMC4539866

[44] Soares CN, Zitek B. Reproductive hormone sensitivity and risk for depression across the female life cycle: a continuum of vulnerability? J Psychiatry Neurosci. 2008;33(4):331-343. 18592034 PMC2440795

[45] Li W, Liu Q, Deng X, Chen Y, Liu S, Story M. Association between obesity and puberty timing: a systematic review and meta-analysis. Int J Environ Res Public Health. 2017;14(10):E1266. doi:10.3390/ijerph14101266 29064384 PMC5664767

[46] Bell JA, Carslake D, Wade KH, . Influence of puberty timing on adiposity and cardiometabolic traits: a mendelian randomisation study. PLoS Med. 2018;15(8):e1002641. doi:10.1371/journal.pmed.1002641 30153260 PMC6112630

[47] Lewis G, Ioannidis K, van Harmelen AL, . The association between pubertal status and depressive symptoms and diagnoses in adolescent females: a population-based cohort study. PLoS One. 2018;13(6):e0198804. doi:10.1371/journal.pone.0198804 29912985 PMC6005470

[48] Timonen M, Rajala U, Jokelainen J, Keinänen-Kiukaanniemi S, Meyer-Rochow VB, Räsänen P. Depressive symptoms and insulin resistance in young adult males: results from the Northern Finland 1966 birth cohort. Mol Psychiatry. 2006;11(10):929-933. doi:10.1038/sj.mp.4001838 16702975

[49] Perry BI, Khandaker GM, Marwaha S, . Insulin resistance and obesity, and their association with depression in relatively young people: findings from a large UK birth cohort. Psychol Med. 2020;50(4):556-565. doi:10.1017/S0033291719000308 30854996 PMC7093318

[50] Cham H, Reshetnyak E, Rosenfeld B, Breitbart W. Full information maximum likelihood estimation for latent variable interactions with incomplete indicators. Multivariate Behav Res. 2017;52(1):12-30. doi:10.1080/00273171.2016.1245600 27834491 PMC5489914

[51] Little TD, Jorgensen TD, Lang KM, Moore EW. On the joys of missing data. J Pediatr Psychol. 2014;39(2):151-162. doi:10.1093/jpepsy/jst048 23836191

