# Supplementary material for: Longitudinal Trends in Childhood Insulin Levels and Body Mass Index and Associations With Risks of Psychosis and Depression in Young Adults
Source: JAMA Psychiatry. 2021 Jan 13;78(4):1–11. doi: 10.1001/jamapsychiatry.2020.4180 (PMC7807390; doi:10.1001/jamapsychiatry.2020.4180)
Supplement: Supplement. — eMethods. Detailed Methods eTable 1. Growth Mixture Model Fit Indices for Fasting Insulin Level eTable 2. Growth Mixture Model Fit Indices for Body Mass Index eTable 3. Odds Ratios for Multinomial Logistic Regression Analyses Examining Predictors of Membership of Fasting Insulin Trajectories eTable 4. Anthropometric and Biochemical Characteristics at Age 24 of Participants included in Fasting Insulin Trajectory Classes eTable 5. Odds Ratios for Metabolic Syndrome at Age 24 Associated with Fasting Insulin and Body Mass Index Trajectories eTable 6. Odds Ratios for Multinomial Regression Analyses Examining Predictors of Membership of Body Mass Index Trajectories eTable 7. Anthropometric and Biochemical Characteristics at Age 24 of Participants included in Body Mass Index Trajectory Classes eTable 8. Odds Ratios for Associations of Fasting Insulin Trajectories with Continuous Psychiatric Outcomes eTable 9. Odds Ratios for Associations of Body Mass Index Trajectories with Continuous Psychiatric Outcomes eTable 10. Odds Ratios for Sex-Stratified Associations of Fasting Insulin Trajectories with Binary Psychiatric Outcomes eTable 11. Odds Ratios for Sex-Stratified Associations of Fasting Insulin Trajectories with Continuous Psychiatric Outcomes eTable 12. Odds Ratios for Sex-Stratified Associations of Body Mass Index Trajectories with Binary Psychiatric Outcomes eTable 13. Odds Ratios for Sex-Stratified Associations of Body Mass Index Trajectories with Continuous Psychiatric Outcomes eFigure 1. Flowchart of Available Sample for Primary Analyses eFigure 2. Trajectory Means and Individual Values per Developmental Trajectory of Fasting Insulin eFigure 3. Sensitivity Analysis Examining Trajectories of Fasting Insulin Between Ages 15-24 eFigure 4. Trajectory Means and Individual Values per Developmental Trajectory of Body Mass Index eReferences [file jamapsychiatry-e204180-s001.pdf]

Supplementary Online Content

Perry BI, Stochl J, Upthegrove R, et al. Longitudinal trends in childhood insulin levels and body mass index and associations with risks of psychosis and depression in young adults. *JAMA Psychiatry*. Published online January 13, 2021. doi:10.1001/jamapsychiatry.2020.4180

eMethods. Detailed Methods

eTable 1. Growth Mixture Model Fit Indices for Fasting Insulin Level

eTable 2. Growth Mixture Model Fit Indices for Body Mass Index

eTable 3. Odds Ratios for Multinomial Logistic Regression Analyses Examining Predictors of Membership of Fasting Insulin Trajectories

eTable 4. Anthropometric and Biochemical Characteristics at Age 24 of Participants included in Fasting Insulin Trajectory Classes

eTable 5. Odds Ratios for Metabolic Syndrome at Age 24 Associated with Fasting Insulin and Body Mass Index Trajectories

eTable 6. Odds Ratios for Multinomial Regression Analyses Examining Predictors of Membership of Body Mass Index Trajectories

eTable 7. Anthropometric and Biochemical Characteristics at Age 24 of Participants included in Body Mass Index Trajectory Classes

eTable 8. Odds Ratios for Associations of Fasting Insulin Trajectories with Continuous Psychiatric Outcomes

eTable 9. Odds Ratios for Associations of Body Mass Index Trajectories with Continuous Psychiatric Outcomes

eTable 10. Odds Ratios for Sex-Stratified Associations of Fasting Insulin Trajectories with Binary Psychiatric Outcomes

eTable 11. Odds Ratios for Sex-Stratified Associations of Fasting Insulin Trajectories with Continuous Psychiatric Outcomes

eTable 12. Odds Ratios for Sex-Stratified Associations of Body Mass Index Trajectories with Binary Psychiatric Outcomes

eTable 13. Odds Ratios for Sex-Stratified Associations of Body Mass Index Trajectories with Continuous Psychiatric Outcomes

eFigure 1. Flowchart of Available Sample for Primary Analyses

eFigure 2. Trajectory Means and Individual Values per Developmental Trajectory of Fasting Insulin

eFigure 3. Sensitivity Analysis Examining Trajectories of Fasting Insulin Between Ages 15-24

eFigure 4. Trajectory Means and Individual Values per Developmental Trajectory of Body Mass Index

eReferences

This supplementary material has been provided by the authors to give readers additional information about their work.

© 2021 Perry BI et al. *JAMA Psychiatry.*

eMethods. Detailed Methods

Assessment of Potential Confounders

Sex was recorded at birth (binary variable). Ethnicity was recorded from participant-completed questionnaire data and coded as White vs. non-White. Paternal social class was recorded from participant-completed questionnaire data based on occupation. Smoking (on average >1 cigarette each day) was coded as a binary variable at 15y, 18y and 24y from participant-completed questionnaire data, and we summed the variables to create a cumulative smoking score between 15-24y of 0-3. Physical activity (averaged over the past year) was recorded from participant-completed questionnaire data at 15y, 18y and 24y as: 0=never, 1=less than once per month; 2=one to three times per month; 3=one to four times per week; 4= five or more times per week. We summed the three variables creating a cumulative physical activity score between 15-24y of 0-12. Alcohol use was coded as a binary variable (>1 alcoholic beverage on average each week) at 12y, 15y, 18y and 24y from participant-completed questionnaire data. We summed the four variables creating a cumulative alcohol use score between 12-24y of 0-4. Substance use was coded as a binary variable at ages 12y, 15y, 18y and 24y. At age 12y, the self-report questionnaire asked whether the participant had ever taken any illicit substance. At ages 15y and 18y, the self-report questionnaire asked whether the participant had taken any illicit substance in the past year. At age 24y, the self-report questionnaire asked how many illicit substances the participant had taken in the past year. We recoded the age 24 variable as a binary variable, with a score of 1 if the participant recorded taking at least one illicit substance in the past year. We summed the four variables creating a cumulative substance use score between 12-24y of 0-4. Sleep problems were coded as binary variables at 7y, 8y, 9y and 14y from questionnaire data completed by the primary caregiver, and at 15y completed by the participant. At 7y and 9y the primary care-giver was asked whether the participant had difficulty sleeping in the past year, and at 8y, 14y and 15y the same question was asked with a duration of the past month. We summed the five variables creating a cumulative sleeping difficulties score between 7-15y of 0-5. Average calorie intake was assessed at 7y, 10y and 13y via a food frequency questionnaire, which was sent to the primary caregiver a week before the child's clinic appointment. The primary caregiver was asked to record everything the child ate or drank for a three-day period, including one weekend day. When they brought the child to the clinic appointment, they were interviewed by a trained member of the nutrition team with the aim of ensuring the completeness of the record with regard to the type of food/drink and the amount eaten/drunk. At each age, average daily calorie (kcal) intake was recorded. We standardized (z-scores) the three variables and summed them together, creating a cumulative average calorie intake score between 7-13y. Childhood emotional and behavioural problems were assessed at age 7y via the Strength and Difficulties Questionnaire[1], which screens for emotional symptoms, hyperactivity/inattention and peer relationship problems, and summed into a 'total difficulties score', which we used as an adjustment variable. However, due to a large reduction in the available sample size when the SDQ 'total difficulties score' was included as an adjustment variable to regression models testing associations of trajectory class membership with psychiatric outcomes, we used the *k*-nearest neighbours (Knn) imputation algorithm of the VIM package[2] in R (using recommended settings) to replace missing data for the SDQ variable only. The Knn algorithm is sensitive and robust to different data types, and performs comparatively well to other methods of imputation such as multiple imputation using chained equations[3,4]. We used Knn imputation in place of multiple imputation methods since where the former produces a single imputed dataset, the latter produces multiple imputed datasets which would have led to significant and prohibitive computation burden coupled with the three-step method of analysis (see below).

Clinical and Biochemical Factors

We examined the clinical phenotype of BMI and FI trajectories at age 24. We assessed mean values of commonly measured clinical and biochemical factors from participants grouped by most-likely trajectory class. We included measures of BMI (weight in kg / height in cm, assessed during clinic assessment), waist circumference (cm, assessed during clinical assessment), fasting plasma glucose, high-density lipoprotein (HDL), low-density lipoprotein (LDL), (all mmol/L), fasting insulin (μIU/mL) and CRP (mg/L). All biochemical samples were taken at 0900 during clinic assessment from consenting participants, following a 10-hour fast (water only). For waist circumference, we present mean values separately for males and females since the reference ranges are different.

Statistical Methods

Growth Mixture Modelling

Z-transformed values for FI and BMI were loaded into MPlus using the *MplusAutomation* package for R[5]. Curvilinear growth mixture modeling (GMM) was run individually for FI and BMI. GMM was done using full information maximum likelihood (FIML) estimation to account for cases with missing data, as FIML estimates parameters directly using all the information that is already contained in the incomplete data set[6]. FIML has demonstrated to produce unbiased estimates[7] and valid model fit information[8]. To determine the optimum number of developmental trajectories, several measures of fit indices and tests of model fit were used in addition to interpretability and theoretical justification. GMM was run iteratively whilst increasing the number of trajectory classes to fit. Estimates of the Bayesian Information Criterion (BIC), entropy, Vuong-Lo-Mendell-Rubin Likelihood Ratio Test (VLMR-LRT) and Parametric Bootstrap Likelihood Ratio Test (BLRT) were recorded at each iteration, along with a visual inspection of graphical outputs. Once achieving successful convergence, checks were performed to rule out local solutions by

© 2021 Perry BI et al. *JAMA Psychiatry.*

replicating the estimation using the same seed values and comparing model parameter estimates for replication. A successfully converged model with no local solutions would have the best loglikelihood values repeated[9]. In selecting the optimum class solution, we aimed to select the solution with the lowest BIC, suitable statistical evidence ($p<0.05$) in VLMR-LRT and BLRT tests (suggesting the solution with *n* trajectories is an improvement over the solution with *n-1* trajectories), high entropy values (close to 1.0), and no less than 1% of the total sample in a particular trajectory (to allow further analysis with adequate sample sizes).

Three-Step Method
After completing the first step described above, the second step is to calculate classification uncertainty, which is computed as a natural log of the average latent class probabilities for most likely class membership and the number of observations per trajectory class. These logits are used in the third step, which includes either regression on predictors of trajectory class membership (using trajectory class membership as an outcome), or regression of trajectory classes on an outcome (using trajectory class membership as a predictor). Detailed information on the statistical methodology underpinning the three-step method alongside data simulations are available elsewhere[10]. Mplus includes two methods with which to proceed with the three-step method of analysis. The first is the automatic method, in which either predictors of trajectory class membership, or outcomes, are added as auxiliary variables in the variable command, and specified as such (for example with the R3STEP option for predictors of trajectory class membership). The automatic method is suitable for simple univariable analysis, and we used this option to examine the associations of sociodemographic and lifestyle factors with trajectory class membership. The automatic method is unsuitable for analyses which include adjustment for confounders, and so the manual method must be used. In the manual method, the most likely trajectory class posterior distribution is obtained using the SAVEDATA command in MPlus, with the option SAVE=CPROB. In step 2, classification uncertainty is computed as a natural log of the average latent class probabilities for most likely class membership and the number of observations per trajectory class. These logits are provided within the Mplus output for step 1. In step 3, the desired model where the latent class variable is measured by the most likely class variable N and the measurement error for each trajectory class is fixed and prespecified to the logits computed in Step 2. In step 3, starting values are set to 0, since class membership was already determined in step 1. We used the manual method for our primary analysis examining the associations between trajectory class membership and psychiatric outcomes, since our analysis included adjusting for potential confounders. Please see an example Mplus scripts below for the manual method for our analysis of BMI trajectories.

Example Script for Step 1 of the Manual 3 Step Method
```
TITLE: BMI Growth Model Ages 1-24 - 3 Step Manual Method – Step 1

DATA: FILE = "zBMIwithaux.dat";
VARIABLE:
NAMES = CaseNo ZBMI_1 ZBMI_2 ZBMI_3 ZBMI_4 ZBMI_7
       ZBMI_9 ZBMI_10 ZBMI_11 ZBMI_12
       ZBMI_15 ZBMI_18 ZBMI_24 Sex Ethnicity
       Social_class cumul_smok cumul_physact sdq_7 cumul_alc cumul_subsuse cumul_sleep depressive_episode24;
MISSING =.;
AUXILIARY = Sex Ethnicity  Social_class class cumul_smok cumul_physact sdq_7 cumul_alc cumul_subsuse
cumul_sleep cumul_kcal depressive_episode24;
IDVARIABLE = CaseNo;
  USEVARIABLES = ZBMI_1 ZBMI_2 ZBMI_3 ZBMI_4 ZBMI_7
ZBMI_9 ZBMI_10 ZBMI_11 ZBMI_12 ZBMI_15 ZBMI_18 ZBMI_24;
  CLASSES = c(5);
  SAVEDATA: FILE = man3step_BMI_step1.dat;
  save = CPROB;
  ANALYSIS:TYPE = mixture;
           STITITERATIONS=20;
               STARTS = 250 25;

 MODEL:
   %OVERALL%
    i s q | ZBMI_1@1 ZBMI_2@2 ZBMI_3@3 ZBMI_4@4 ZBMI_7@7
ZBMI_9@9 ZBMI_10@10 ZBMI_11@11 ZBMI_12@12
     ZBMI_15@15 ZBMI_18@18 ZBMI_24@24;
%c#1%
i s;
%c#2%
i s;
%c#3%
i s;
```

© 2021 Perry BI et al. *JAMA Psychiatry.*

```
%c#4%
i s;
%c#5%
i s;
   Output: sampstat standardized
   tech4 tech11 tech14;
   CINTERVAL
```

Sociodemographic and Lifestyle Associations of Trajectories

Time Invariant Factors

Sex at birth was coded as a binary variable (male / female). Ethnicity was coded as a binary variable (white vs non-white), assessed via questionnaire data. Social class at birth was defined based upon father's occupation as per the UK Office of National Statistics classification system: I, II, III non-manual, III manual, IV, V). We coded a binary variable of 'lower social class' with "1" given to social class classification < III. A positive family history of cardiometabolic/cardiovascular disease was coded from self-report questionnaire data encompassing hypertension, T2DM, hypercholesterolaemia, or cardiovascular diseases. Stressful life events (SLEs) were based on self-report questionnaire data comprising a summed total of up to 42 pre-specified life events affecting the mother at 18- and 36-weeks gestation, and affecting the participant at 8-weeks and 6-months postpartum. Examples included death or loss of a partner or family member, loss of employment, moving-house or financial difficulty. A full list of the 42 SLEs is reported elsewhere[11]. We compared the top tertile of summed SLE scores vs the bottom tertile. Birthweight and gestational age were coded as continuous variables derived from questionnaire data.

Time Variant Factors

Low exercise at ages 15 and 18 years was coded from self-report questionnaire data and defined as participating in any physical activity less than once per week on average in the past year. Smoking at ages 15 and 18 years was coded from self-report questionnaire data and defined as smoking on average >1 cigarette per day.

Clinical and Biochemical Phenotype of Trajectory Classes at Age 24

BMI was assessed during clinic assessment, from measures of height (m^2) and weight (kg). Waist circumference was measured during clinic assessment. Blood-based samples (fasting plasma glucose (FPG), fasting insulin (FI), high-density lipoprotein, low-density lipoprotein, triglycerides and C-reactive protein) were after an 8-hour fast (water only). Samples were immediately spun, frozen and stored at –80ºC and measurements were assayed within 3 to 9 months of the samples being taken with no previous freeze-thaw cycles.

Association of Trajectory Membership with Metabolic Syndrome at Age 24

Metabolic syndrome was defined[12] as the presence of ethnicity-specific waist circumference (≥94cm in males and ≥80cm in females for caucasians; ≥90cm in males and ≥80cm in females in other ethnic groups[12]) or raised BMI (>29.9), plus two from; elevated triglycerides (≥150mg/dL); reduced HDL (male <40mg/dL; female <50mg/dL); elevated systolic blood pressure (≥130mmHg) or elevated fasting plasma glucose (FPG) (≥100mg/dL). Logistic regression via the three-step method[10] was used to examine the association of trajectory membership for FI and BMI with metabolic syndrome at age 24, compared with the most common trajectory, before and after adjusting for the same confounders used in the primary analysis.

© 2021 Perry BI et al. *JAMA Psychiatry.*

eTable 1: Growth Mixture Model Fit Indices for Fasting Insulin Level

***n* Trajectories**	**BIC**	**Entropy**	**VLMR-LRT (*p*-value)**	**BLRT (*p*-value)**
1	76474	-	-	-
2	69389	0.957	0.007	<0.001
3†	66304	0.853	0.03	<0.001
4*	67872	0.750	0.25	0.04
5*	67688	0.836	0.32	0.11
6*	67521	0.729	0.41	0.44

BIC = Bayesian Information Criterion; VLMR-LRT = Vuong-Lo-Mendell-Rubin Likelihood Ratio Test; BLRT = Parametric Bootstrap Likelihood Ratio Test

**Contained one trajectory with <1% of sample*

†Selected for further analysis

© 2021 Perry BI et al. *JAMA Psychiatry.*

eTable 2: Growth Mixture Model Fit Indices for Body Mass Index

***n* Trajectories**	**BIC**	**Entropy**	**VLMR-LRT (*p*-value)**	**BLRT (*p*-value)**
1	223514	-	-	-
2	224574	0.663	<0.001	<0.001
3	222745	0.774	<0.001	<0.001
4	222142	0.768	0.03	<0.001
5[†]	221575	0.885	0.01	<0.001
6*	221138	0.766	0.10	0.07

BIC = Bayesian Information Criterion; VLMR-LRT = Vuong-Lo-Mendell-Rubin Likelihood Ratio Test; BLRT = Parametric Bootstrap Likelihood Ratio Test

**Contained one trajectory with <1% of sample*

[†]Selected for further analysis

© 2021 Perry BI et al. *JAMA Psychiatry.*

eTable 3: Odds Ratios for Multinomial Logistic Regression Analyses Examining Predictors of Membership of Fasting Insulin Trajectories

Variable	Odds Ratio (95% CI)		
	Class 1[a] 'Stable Average'	Class 2 'Minor Increase'	Class 3 'Persistently High'
Female Sex	1.00	1.37 (1.10-2.04)	1.10 (0.89-1.23)
Non-WB Ethnicity	1.00	1.22 (0.89-1.62)	1.21 (0.91-1.73)
Lower Social Class (>3)	1.00	1.05 (1.00-1.09)	1.89 (1.35-2.50)
FHx CMet	1.00	1.10 (0.92-1.41)	1.66 (1.14-1.69)
Gestational Age	1.00	1.10 (0.95-1.31)	1.21 (0.90-1.44)
Birthweight	1.00	0.89 (0.60-1.10)	0.76 (0.44-0.92)
Stressful Life Events (Top tertile)	1.00	1.21 (0.55-4.32)	2.06 (1.43-4.31)
Low Exercise (age 15)	1.00	1.13 (1.06-1.31)	1.16 (1.02-1.41)
Smoking (age 15)	1.00	1.45 (1.03-1.76)	1.10 (0.86-1.55)
Low Exercise (age 18)	1.00	1.45 (1.14-1.89)	1.54 (1.06-2.22)
Smoking (age 18)	1.00	1.39 (1.07-1.43)	1.40 (1.10-1.78)

© 2021 Perry BI et al. *JAMA Psychiatry.*

eTable 4: Anthropometric and Biochemical Characteristics at Age 24 of Participants included in Fasting Insulin Trajectory Classes

Measure, Mean (SD)	Trajectory		
	Class 1 (Stable Average)	Class 2 (Minor Increase)	Class 3 (Persistently High)
Fasting Insulin (μIU/mL)	6.93 (2.70)	8.57 (1.21)*	13.65 (4.32)*
Body Mass Index (kg/m^2)	22.12 (3.76)	26.18 (4.23)*	24.76 (7.74)
Waist Circumference, Males (cm)	84.58 (8.79)	99.56 (15.34)	94.45 (16.72)
Waist Circumference, Females (cm)	75.62 (9.44)	91.41 (14.14)*	89.20 (18.14)*
Fasting Plasma Glucose (mgl/dL)	94.41 (12.07)	98.92 (11.71)	104.14 (14.05)
HDL Cholesterol (mgl/dL)	61.78 (15.83)	50.97 (14.67)*	50.58 (17.76)*
Triglycerides (mgl/dL)	78.76 (33.63)	115.93 (77.88)	154.87 (89.38)*
LDL Cholesterol (mg/dL)	92.28 (28.19)	104.63 (30.89)	105.41 (34.36)
C-Reactive Protein (mg/dL)	0.18 (0.40)	0.22 (0.32)	0.34 (0.42)*

All measures presented using Conventional Units. For conversion to SI Units: Fasting insulin (pmol/L) x 6.945); Fasting Plasma Glucose (mmol/L) x 0.0555; Triglycerides (mmol/L) x 0.0113; HDL and LDL Cholesterol (mmol/L) x 0.0259; CRP (mg/L) x 10.
*indicates outside of U.K. reference/"normal" range: Body Mass Index=18.5-24.9kg/m^2 ; Fasting Insulin=3-8μIU/mL; Waist Circumference (males)=<102cm; Waist Circumference (females)=<88cm; Fasting Plasma Glucose=<126mg/dL; HDL=>52mg/dL; Triglycerides=<150mg/dL; LDL=<130mg/dL; CRP<0.3mg/dL.

© 2021 Perry BI et al. *JAMA Psychiatry.*

eTable 5: Odds Ratios for Metabolic Syndrome at Age 24 Associated with Fasting Insulin and Body Mass Index Trajectories

Trajectory	Sample	Odds Ratio (95% C.I.)		*p*-value
		Unadjusted	Adjusted for sex, ethnicity, social class, SDQ (7y), cumulative smoking, physical activity, alcohol and substance use, sleep and calorie intake	
**Fasting Insulin**				
Class 1 – ‘Stable Average’	4,939	1.00 [reference]	1.00 [reference]	-
Class 2 – ‘Minor Increase’	693	5.14 (3.01-8.09)	4.24 (2.34-8.21)	<0.001
Class 3 – ‘Persistently High’	158	10.51 (4.82-22.18)	9.21 (3.77-20.15)	<0.001
**BMI**				
Class 1 – ‘Stable Average’	8,383	1.00 [reference]	1.00 [reference]	-
Class 2 – ‘Gradually Decreasing’	949	0.91 (0.57-1.48)	0.90 (0.55-1.46)	0.68
Class 3 – ‘Puberty Onset – Minor Increase’	668	6.02 (4.32-8.38)	5.64 (3.31-9.01)	<0.001
Class 4 – ‘Puberty Onset – Major Increase’	174	7.80 (3.67-13.54)	6.91 (3.20-12.87)	<0.001
Class 5 – ‘Persistently High	289	11.65 (7.45-15.45)	10.62 (5.89-19.13)	<0.001

© 2021 Perry BI et al. *JAMA Psychiatry.*

eTable 6: Odds Ratios for Multinomial Regression Analyses Examining Predictors of Membership of Body Mass Index Trajectories

Variable	Odds Ratio (95% CI)				
	Class 1[a] 'Stable Average'	Class 2 'Gradually Decreasing'	Class 3 'Puberty Onset – Minor Increase'	Class 4 'Puberty Onset – Major Increase'	Class 5 'Persistently High'
Female Sex	1.00	1.10 (0.90-1.21)	1.35 (1.12-1.56)	1.10 (0.91-1.26)	0.89 (0.70-1.13)
Non-WB Ethnicity	1.00	1.76 (1.16-2.65)	1.09 (0.61-1.93)	1.12 (0.35-3.56)	0.62 (0.20-1.96)
Lower Social Class	1.00	1.08 (0.99-1.17)	1.11 (1.01-1.21)	1.13 (1.05-1.22)	1.26 (1.11-1.40)
FHx CMet	1.00	1.19 (0.92-1.55)	1.48 (1.20-1.84)	2.43 (1.35-4.37)	2.69 (1.82-3.98)
Gestational Age	1.00	0.98 (0.92-1.06)	1.00 (0.94-1.07)	1.12 (0.43-2.95)	1.12 (0.87-1.32)
Birthweight	1.00	1.30 (1.18-1.43)	0.99 (0.93-1.03)	0.90 (0.83-1.15)	1.44 (1.25-1.65)
Stressful Life Events	1.00	0.84 (0.68-1.04)	1.11 (0.96-1.30)	1.44 (1.01-2.07)	1.89 (1.44-3.21)
Low Exercise (age 15)	1.00	1.06 (0.84-1.32	1.38 (1.13-1.69)	1.90 (1.08-3.35)	1.36 (0.87-2.12)
Smoking (age 15)	1.00	1.22 (0.75-2.03)	1.62 (1.17-2.25)	1.14 (0.57-3.60)	1.20 (0.72-2.01)
Low Exercise (age 18)	1.00	0.78 (0.56-0.95)	1.31 (1.04-1.65)	1.50 (1.01-2.90)	0.94 (0.65-1.36)
Smoking (age 18)	1.00	1.15 (0.71-1.86)	1.63 (1.12-2.38)	2.37 (0.99-5.72)	1.44 (0.73-2.84)

[a]reference group

© 2021 Perry BI et al. *JAMA Psychiatry.*

eTable 7: Anthropometric and Biochemical Characteristics at Age 24 of Participants included in Body Mass Index Trajectory Classes

Biochemical and Anthropometric Variable, Mean (SD)	Trajectory				
	Class 1 (Stable Average)	Class 2 (Gradually Decreasing)	Class 3 (Puberty Onset – Minor Increase)	Class 4 (Puberty Onset – Major Increase)	Class 5 (Persistently High)
Body Mass Index	23.60 (3.46)	25.32 (3.85)*	27.25 (4.47)*	33.67 (8.68)*	31.55 (5.66)*
Fasting Insulin (μIU/mL)	6.42 (2.12)	6.45 (3.39)	7.32 (4.44)	8.44 (5.43)*	8.21 (3.19)*
Waist Circumference Males (cm)	83.50 (8.45)	86.40 (10.36)	100.67 (11.60)	121.46 (6.70)*	111.77 (12.29)*
Waist Circumference Females (cm)	75.62 (9.12)	78.80 (10.15)	87.12 (11.71)	99.76 (19.17)*	94.97 (14.26)*
Fasting Plasma Glucose (mg/dL)	95.14 (12.61)	94.41 (8.83)	98.02 (10.63)	95.58 (9.19)	98.92 (17.48)
HDL Cholesterol (mg/dL)	60.62 (16.22)	58.69 (16.22)	55.98 (12.36)	50.97 (8.11)	52.12 (17.76)
Triglycerides (mg/dL)	83.19 (44.25)	82.30 (41.59)	118.58 (72.57)	127.43 (0.61)	53.98 (68.14)
LDL Cholesterol (mg/dL)	93.05 (28.96)	91.51 (28.19)	95.75 (30.89)	106.95 (23.94)	107.72 (33.59)
C-Reactive Protein (mg/dL)	0.21 (0.69)	0.21 (0.40)	0.30 (0.45)*	0.48 (0.38)*	0.40 (0.42)*

All measures presented using Conventional Units. For conversion to SI Units: Fasting insulin (pmol/L) x 6.945); Fasting Plasma Glucose (mmol/L) x 0.0555; Triglycerides (mmol/L) x 0.0113; HDL and LDL Cholesterol (mmol/L) x 0.0259; CRP (mg/L) x 10.

*indicates outside of U.K. reference/"normal" range: Body Mass Index=18.5-24.9kg/m^2 ; Fasting Insulin=3-8μIU/mL; Waist Circumference (males)=<102cm; Waist Circumference (females)=<88cm; Fasting Plasma Glucose=<126mg/dL; HDL=>50mg/dL; Triglycerides=<150mg/dL; LDL=<130mg/dL; CRP<0.3mg/dL.

© 2021 Perry BI et al. *JAMA Psychiatry.*

eTable 8: Odds Ratios for Associations of Fasting Insulin Trajectories with Continuous Psychiatric Outcomes

Trajectory	Sample	Beta Coefficient (95% C.I.)		*p*-value[a]
		Unadjusted	Adjusted for sex, ethnicity, social class, SDQ (7y), cumulative smoking, physical activity, alcohol and substance use, sleep, and calorie intake , negative/depressive symptoms	
**Depressive Symptom Score at Age 24**				
Class 1 – 'Stable Average'	4,939	0.00 [reference]	0.00 [reference]	-
Class 2 – 'Minor Increase'	693	0.03 (-0.02, 0.08)	0.02 (-0.04. 0.08)	1.00
Class 3 – 'Persistently High'	158	0.08 (0.04, 0.13)	0.05 (-0.03,0.13)	0.67
**Negative Psychotic Symptom Score at Age 24**				
Class 1 – 'Stable Average'	4,939	0.00 [reference]	0.00 [reference]	-
Class 2 – 'Minor Increase'	693	0.08 (-0.01,0.16)	0.05 (0.01,0.09)	0.19
Class 3 – 'Persistently High'	158	0.18 (0.10,0.26)	0.07 (0.01, 0.13)	0.05

[a]*p*-values adjusted for multiple testing using Holm-Bonferroni method

© 2021 Perry BI et al. *JAMA Psychiatry.*

eTable 9: Odds Ratios for Associations of Body Mass Index Trajectories with Continuous Psychiatric Outcomes

Trajectory	Sample	Beta Coefficient (95% C.I.)		*p*-value[a]
		Unadjusted	Adjusted for sex, ethnicity, social class, SDQ (7y), cumulative smoking, physical activity, alcohol and substance use, sleep and calorie intake negative/depressive symptoms	
**Depressive Symptom Score at Age 24**				
Class 1 – 'Stable Average'	8,383	0.00 [reference]	0.00 [reference]	-
Class 2 – 'Gradually Decreasing'	949	0.02 (-0.06, 0.10)	0.01 (-0.05, 0.08)	1.00
Class 3 – 'Puberty Onset – Minor Increase'	668	0.14 (0.08, 0.20)	0.06 (0.01, 0.11)	0.12
Class 4 – 'Puberty Onset – Major Increase'	174	0.20 (0.10, 0.31)	0.08 (0.03, 0.14)	0.03
Class 5 – 'Persistently High	289	0.10 (-0.09, 0.21)	0.02 (-0.08, 0.13)	1.00
**Negative Psychotic Symptom Score at Age 24**				
Class 1 – 'Stable Average'	8,383	0.00 [reference]	0.00 [reference]	-
Class 2 – 'Gradually Decreasing'	949	0.07 (-0.03, 0.16)	0.04 (-0.05, 0.13)	1.00
Class 3 – 'Puberty Onset – Minor Increase'	668	0.11 (0.05, 0.17)	0.03 (-0.05, 0.11)	0.80
Class 4 – 'Puberty Onset – Major Increase'	174	0.18 (0.11, 0.24)	0.06 (-0.03, 0.16)	0.52
Class 5 – 'Persistently High	289	0.13 (0.02, 0.24)	0.09 (-0.04, 0.23)	1.00

[a]*p*-values adjusted for multiple testing using Holm-Bonferroni method

© 2021 Perry BI et al. *JAMA Psychiatry.*

eTable 10: Odds Ratios for Sex-Stratified Associations of Fasting Insulin Trajectories with Binary Psychiatric Outcomes

Trajectory	Sample	Odds Ratio (95% C.I.)		*p*-value[a]
		Unadjusted	Adjusted for sex, ethnicity, social class, SDQ (7y), cumulative smoking, physical activity, alcohol and substance use, sleep and calorie intake	
**Definite PEs at Age 24 (Males)**				
Class 1 – 'Stable Average'	2,319	1.00 [reference]	1.00 [reference]	-
Class 2 – 'Minor Increase'	278	1.51 (0.91-2.54)	1.01 (0.55-1.83)	1.00
Class 3 – 'Persistently High'	66	1.91 (1.02-5.03)	1.82 (0.67-4.82)	0.47
**Definite PEs at Age 24 (Females)**				
Class 1 – 'Stable Average'	2,620	1.00 [reference]	1.00 [reference]	-
Class 2 – 'Minor Increase'	415	1.32 (1.11-1.89)	1.19 (0.66-2.10)	1.00
Class 3 – 'Persistently High'	92	1.65 (1.12-2.01)	1.22 (0.70-2.15)	1.00
**Psychosis At Risk Mental State at Age 24 (Males)**				
Class 1 – 'Stable Average'	2,319	1.00 [reference]	1.00 [reference]	-
Class 2 – 'Minor Increase'	278	1.65 (0.42-5.30)	1.44 (0.15-13.92)	1.00
Class 3 – 'Persistently High'	66	8.32 (3.13-16.49)	4.48 (1.84-10.91)	0.006
**Psychosis At Risk Mental State at Age 24 (Females)**				
Class 1 – 'Stable Average'	2,620	1.00 [reference]	1.00 [reference]	-
Class 2 – 'Minor Increase'	415	1.39 (0.29-5.57)	1.06 (0.22-5.11)	1.00
Class 3 – 'Persistently High'	92	4.84 (0.47-31.18)	2.99 (0.46-18.37)	0.84
**Psychotic Disorder at Age 24 (Males)**				
Class 1 – 'Stable Average'	2,319	1.00 [reference]	1.00 [reference]	-
Class 2 – 'Minor Increase'	278	1.55 (0.61-4.31)	1.26 (0.49-3.04)	1.00
Class 3 – 'Persistently High'	66	5.79 (1.24-27.09)	3.94 (1.37-11.34)	0.05
**Psychotic Disorder at Age 24 (Females)**				
Class 1 – 'Stable Average'	2,620	1.00 [reference]	1.00 [reference]	-
Class 2 – 'Minor Increase'	415	1.45 (0.63-3.35)	1.42 (0.60-3.31)	1.00

© 2021 Perry BI et al. *JAMA Psychiatry.*

Class 3 – 'Persistently High'	92	3.29 (0.53-9.86)	2.50 (0.57-11.09)	1.00
**Depressive Episode at Age 24 (Males)**				
Class 1 – 'Stable Average'	2,319	1.00 [reference]	1.00 [reference]	-
Class 2 – 'Minor Increase'	278	1.80 (1.04-3.11)	1.33 (0.82-2.24)	1.00
Class 3 – 'Persistently High'	66	0.97 (0.23-4.13)	0.95 (0.22-4.12)	1.00
**Depressive Episode at Age 24 (Females)**				
Class 1 – 'Stable Average'	2,620	1.00 [reference]	1.00 [reference]	-
Class 2 – 'Minor Increase'	415	1.23 (0.88-1.73)	1.17 (0.83-1.66)	1.00
Class 3 – 'Persistently High'	92	1.61 (0.82-3.14)	1.50 (0.76-2.96)	1.00

[a]*p*-values adjusted for multiple testing using Holm-Bonferroni method

© 2021 Perry BI et al. *JAMA Psychiatry.*

eTable 11: Odds Ratios for Sex-Stratified Associations of Fasting Insulin Trajectories with Continuous Psychiatric Outcomes

Trajectory	Sample	Beta Coefficient (95% C.I.)		*p*-value[a]
		Unadjusted	Adjusted for sex, ethnicity, social class, SDQ (7y), cumulative smoking, physical activity, alcohol and substance use, sleep and calorie intake, negative/depressive symptoms	
**Depressive Symptom Score at Age 24 (Males)**				
Class 1 – 'Stable Average'	2,319	0.00 [reference]	0.00 [reference]	-
Class 2 – 'Minor Increase'	278	0.03 (-0.05, 0.08)	0.01 (-0.09, 0.10)	1.00
Class 3 – 'Persistently High'	66	0.10 (-0.12, 0.23)	0.03 (-0.04, 0.10)	1.00
**Depressive Symptom Score at Age 24 (Females)**				
Class 1 – 'Stable Average'	2,620	0.00 [reference]	0.00 [reference]	-
Class 2 – 'Minor Increase'	415	0.05 (-0.04, 0.09)	0.00 (-0.05, 0.06)	1.00
Class 3 – 'Persistently High'	92	0.06 (-0.08, 0.15)	0.02 (-0.09, 0.14)	1.00
**Negative Psychotic Symptom Score at Age 24 (Males)**				
Class 1 – 'Stable Average'	2,319	0.00 [reference]	0.00 [reference]	-
Class 2 – 'Minor Increase'	278	0.11 (0.02, 0.24)	0.08 (0.02, 0.15)	0.04
Class 3 – 'Persistently High'	66	0.23 (0.08, 0.38)	0.12 (0.03, 0.21)	0.02
**Negative Psychotic Symptom Score at Age 24 (Females)**				
Class 1 – 'Stable Average'	2,620	0.00 [reference]	0.00 [reference]	-
Class 2 – 'Minor Increase'	415	0.08 (0.03, 0.14)	0.04 (0.00, 0.07)	0.25
Class 3 – 'Persistently High'	92	0.15 (-0.03, 0.32)	0.03 (-0.04, 0.10)	0.59

[a]*p*-values adjusted for multiple testing using Holm-Bonferroni method

© 2021 Perry BI et al. *JAMA Psychiatry.*

eTable 12: Odds Ratios for Sex-Stratified Associations of Body Mass Index Trajectories with Binary Psychiatric Outcomes

Trajectory	Sample	Odds Ratio (95% C.I.)		*p*-value[a]
		Unadjusted	Adjusted for sex, ethnicity, social class, SDQ (7y), cumulative smoking, physical activity, alcohol and substance use, sleep and calorie intake	
**Definite PEs at Age 24 (Males)**				
Class 1 – 'Stable Average'	4,164	1.00 [reference]	1.00 [reference]	-
Class 2 – 'Gradually Decreasing'	443	1.22 (0.46-1.87)	0.76 (0.37-1.55)	1.00
Class 3 – 'Puberty Onset – Minor Increase'	311	1.22 (0.63-2.36)	0.62 (0.19-1.98)	1.00
Class 4 – 'Puberty Onset – Major Increase'	105	5.87 (0.53-9.21)	3.22 (0.74-12.55)	1.00
Class 5 – 'Persistently High	107	1.47 (0.43-4.98)	1.28 (0.65-2.44)	1.00
**Definite PEs at Age 24 (Females)**				
Class 1 – 'Stable Average'	4,219	1.00 [reference]	1.00 [reference]	-
Class 2 – 'Gradually Decreasing'	506	1.14 (0.74-1.75)	1.48 (0.92-2.38)	0.50
Class 3 – 'Puberty Onset – Minor Increase'	357	1.90 (1.11-3.26)	1.65 (0.99-2.62)	0.33
Class 4 – 'Puberty Onset – Major Increase'	184	1.54 (0.65-3.66)	0.81 (0.24-2.77)	1.00
Class 5 – 'Persistently High	67	2.32 (0.88-6.13)	1.79 (0.90-3.49)	0.18
**Psychosis At Risk Mental State at Age 24 (Males)**				
Class 1 – 'Stable Average'	4,164	1.00 [reference]	1.00 [reference]	-
Class 2 – 'Gradually Decreasing'	443	0.60 (0.44-2.12)	0.73 (0.31-1.84)	1.00
Class 3 – 'Puberty Onset – Minor Increase'	311	1.51 (0.55-4.64)	1.22 (0.61-2.39)	1.00
Class 4 – 'Puberty Onset – Major Increase'	105	3.13 (1.01-5.12)	2.21 (0.81-5.65)	1.00
Class 5 – 'Persistently High	107	1.69 (0.60-2.01)	1.31 (0.39-4.87)	1.00
**Psychosis At Risk Mental State at Age 24 (Females)**				
Class 1 – 'Stable Average'	4,219	1.00 [reference]	1.00 [reference]	-
Class 2 – 'Gradually Decreasing'	506	0.61 (0.14-2.14)	0.68 (0.19-2.89)	1.00
Class 3 – 'Puberty Onset – Minor Increase'	357	0.76 (0.76-3.21)	0.86 (0.32-2.62)	1.00
Class 4 – 'Puberty Onset – Major Increase'	184	1.81 (0.25-6.43)	1.41 (0.28-5.43)	1.00
Class 5 – 'Persistently High	67	1.21 (0.77-3.21)	1.09 (0.31-4.88)	1.00

© 2021 Perry BI et al. *JAMA Psychiatry.*

**Psychotic Disorder at Age 24 (Male)**				
Class 1 – 'Stable Average'	4,164	1.00 [reference]	1.00 [reference]	-
Class 2 – 'Gradually Decreasing'	443	0.76 (0.54-2.01)	1.08 (0.23-5.01)	1.00
Class 3 – 'Puberty Onset – Minor Increase'	311	1.02 (0.65-1.43)	0.92 (0.21-4.76)	1.00
Class 4 – 'Puberty Onset – Major Increase'	105	2.12 (0.91-4.12)	1.62 (0.71-3.98)	1.00
Class 5 – 'Persistently High	107	3.52 (0.44-15.09)	2.25 (0.62-10.12)	1.00
**Psychotic Disorder at Age 24 (Female)**				
Class 1 – 'Stable Average'	4,219	1.00 [reference]	1.00 [reference]	-
Class 2 – 'Gradually Decreasing'	506	0.40 (0.09-1.21)	0.60 (0.10-3.87)	1.00
Class 3 – 'Puberty Onset – Minor Increase'	357	3.16 (1.29-5.12)	1.88 (0.70-5.06)	1.00
Class 4 – 'Puberty Onset – Major Increase'	184	1.31 (0.65-3.21)	2.60 (0.66-8.21)	1.00
Class 5 – 'Persistently High	67	1.21 (0.40-6.21)	2.74 (0.62-12.22)	1.00
**Depressive Episode at Age 24 (Males)**				
Class 1 – 'Stable Average'	4,164	1.00 [reference]	1.00 [reference]	-
Class 2 – 'Gradually Decreasing'	443	1.31 (0.71-2.44)	1.31 (0.67-2.55)	1.00
Class 3 – 'Puberty Onset – Minor Increase'	311	1.62 (0.83-3.17)	1.60 (0.76-3.36)	1.00
Class 4 – 'Puberty Onset – Major Increase'	105	3.21 (0.67-8.21)	2.23 (0.41-12.72)	1.00
Class 5 – 'Persistently High	107	1.31 (0.30-5.67)	1.77 (0.65-4.39)	1.00
**Depressive Episode at Age 24 (Females)**				
Class 1 – 'Stable Average'	4,219	1.00 [reference]	1.00 [reference]	-
Class 2 – 'Gradually Decreasing'	506	1.20 (0.82-1.85)	1.35 (0.90-2.01)	1.00
Class 3 – 'Puberty Onset – Minor Increase'	357	1.91 (1.15-2.91)	1.52 (1.08-2.29)	0.05
Class 4 – 'Puberty Onset – Major Increase'	184	5.21 (2.09-8.21)	6.28 (2.14-18.44)	0.006
Class 5 – 'Persistently High	67	1.73 (0.86-3.51)	1.94 (0.83-4.67)	1.00

[a]*p*-values adjusted for multiple testing using Holm-Bonferroni method

© 2021 Perry BI et al. *JAMA Psychiatry.*

eTable 13: Odds Ratios for Sex-Stratified Associations of Body Mass Index Trajectories with Continuous Psychiatric Outcomes

Trajectory	Sample	Beta Coefficient (95% C.I.)		*p*-value[a]
		Unadjusted	Adjusted for sex, ethnicity, social class, SDQ (7y), cumulative smoking, physical activity, alcohol and substance use, sleep and calorie intake, negative/depressive symptoms	
**Depressive Symptom Score at Age 24 (Males)**				
Class 1 – 'Stable Average'	4,164	0.00 [reference]	0.00 [reference]	-
Class 2 – 'Gradually Decreasing'	443	0.05 (-0.11, 0.21)	0.01 (-0.19, 0.22)	1.00
Class 3 – 'Puberty Onset – Minor Increase'	311	-0.05 (-0.15, 0.20)	-0.02 (-0.10, 0.12)	1.00
Class 4 – 'Puberty Onset – Major Increase'	105	0.08 (-0.16, 0.23)	0.03 (-0.08, 0.12)	1.00
Class 5 – 'Persistently High	107	0.11 (-0.09, 0.21)	0.03 (-0.07, 0.11)	1.00
**Depressive Symptom Score at Age 24 (Females)**				
Class 1 – 'Stable Average'	4,219	0.00 [reference]	0.00 [reference]	-
Class 2 – 'Gradually Decreasing'	506	0.02 (-0.06, 0.11)	0.01 (-0.10, 0.12)	1.00
Class 3 – 'Puberty Onset – Minor Increase'	357	0.09 (0.02, 0.19)	0.06 (0.03, 0.09)	0.05
Class 4 – 'Puberty Onset – Major Increase'	184	0.15 (0.04, 0.26)	0.09 (0.04, 0.15)	0.05
Class 5 – 'Persistently High	67	0.18 (-0.08, 0.44)	0.03 (-0.07, 0.17)	1.00
**Negative Symptom Score at Age 24 (Males)**				
Class 1 – 'Stable Average'	4,164	0.00 [reference]	0.00 [reference]	-
Class 2 – 'Gradually Decreasing'	443	0.10 (-0.12, 0.33)	0.05 (-0.12, 0.31)	1.00
Class 3 – 'Puberty Onset – Minor Increase'	311	0.13 (0.08, 0.19)	0.04 (-0.03, 0.11)	1.00
Class 4 – 'Puberty Onset – Major Increase'	105	0.21 (-0.12, 0.54)	0.12 (-0.13, 0.36)	1.00
Class 5 – 'Persistently High	107	0.17 (-0.07, 0.42)	0.04 (-0.06, 0.15)	1.00
**Negative Symptom Score at Age 24 (Females)**				
Class 1 – 'Stable Average'	4,219	0.00 [reference]	0.00 [reference]	-
Class 2 – 'Gradually Decreasing'	506	-0.03 (-0.10, 0.16)	0.01 (-0.09, 0.11)	1.00
Class 3 – 'Puberty Onset – Minor Increase'	357	0.07 (0.00, 0.13)	0.03 (-0.07, 0.13)	1.00
Class 4 – 'Puberty Onset – Major Increase'	184	0.16 (0.02, 0.30)	0.04 (-0.09, 0.18)	1.00
Class 5 – 'Persistently High	67	0.11 (-0.05, 0.27)	0.04 (-0.09, 0.17)	1.00

[a]*p*-values adjusted for multiple testing using Holm-Bonferroni method

© 2021 Perry BI et al. *JAMA Psychiatry.*

eFigure 1: Flowchart of Available Sample for Primary Analyses

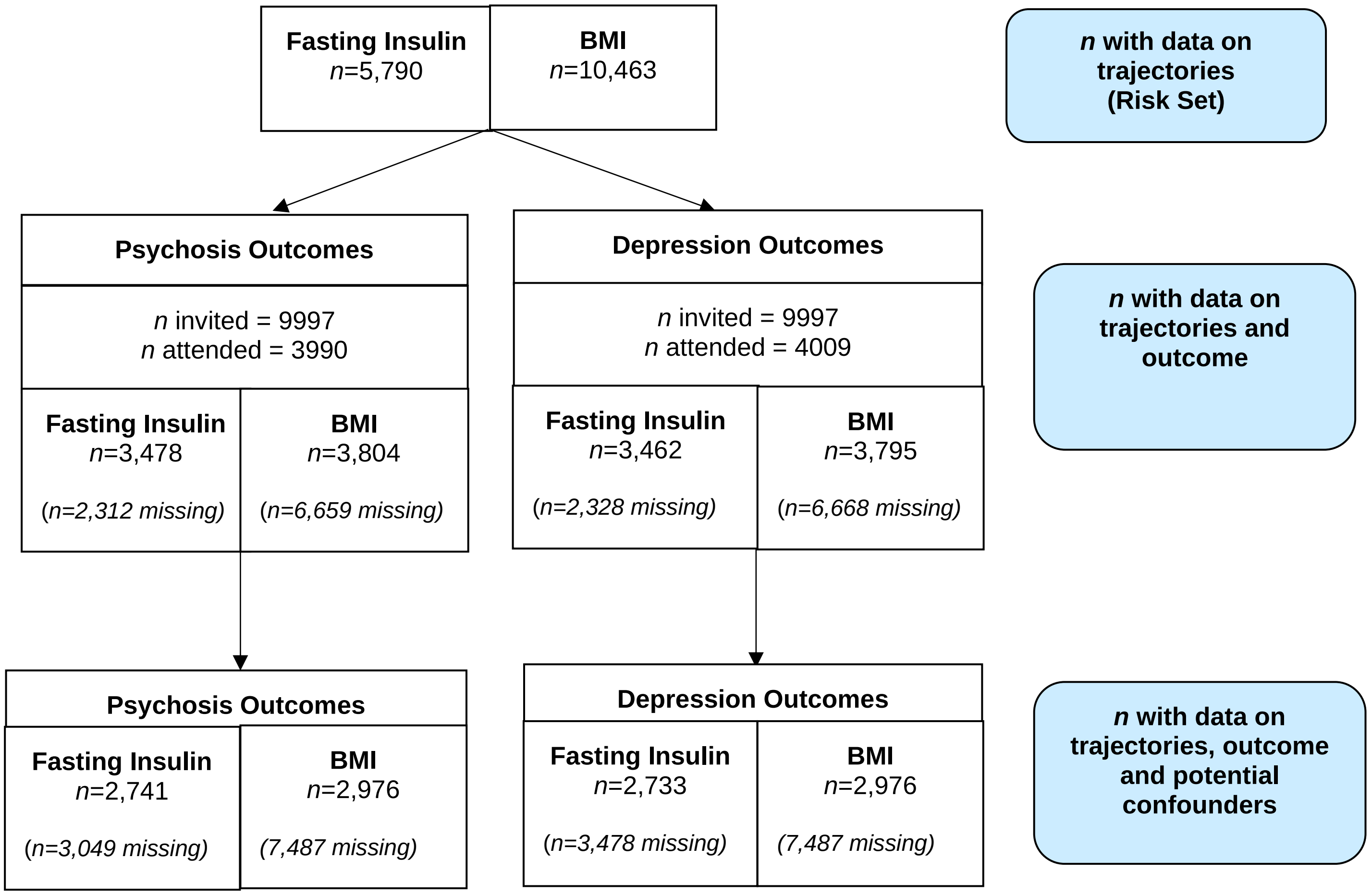

© 2021 Perry BI et al. *JAMA Psychiatry.*

eFigure 2: Trajectory Means and Individual Values per Developmental Trajectory of Fasting Insulin

A. **Class 1 – 'Stable Average'**

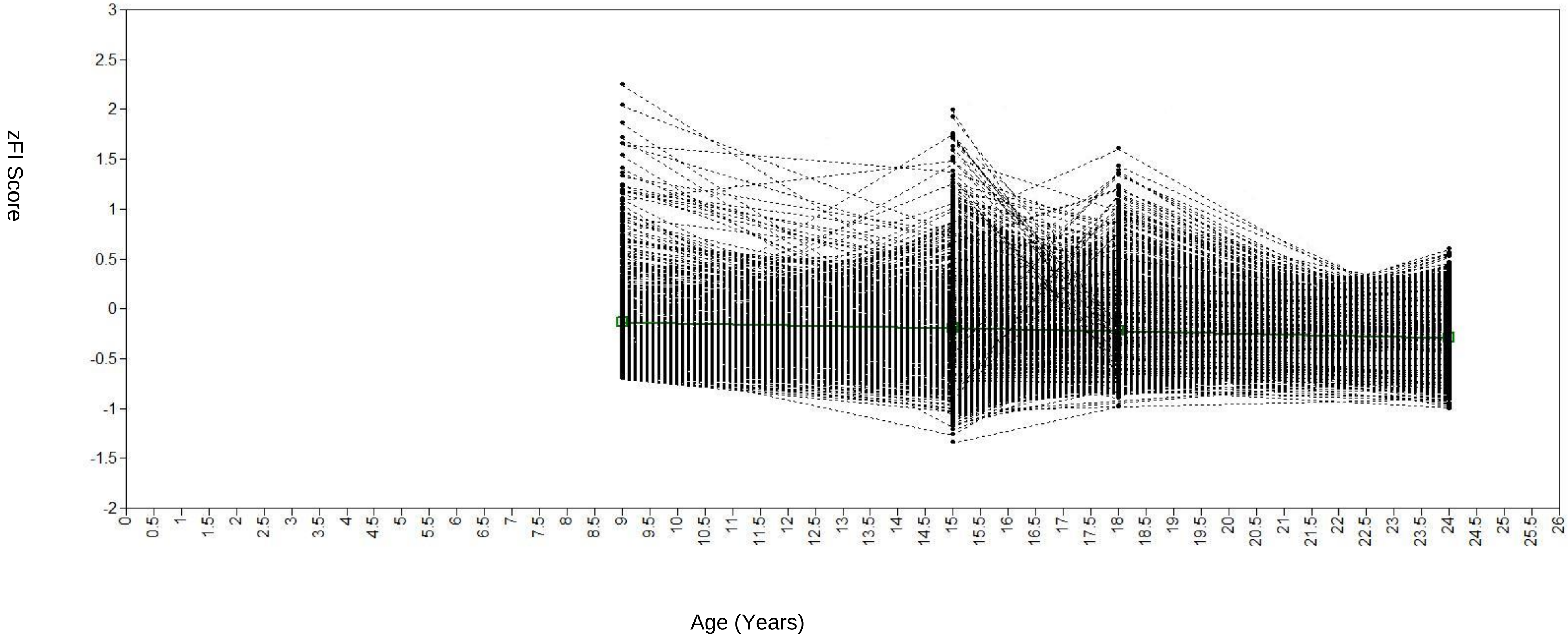

© 2021 Perry BI et al. *JAMA Psychiatry.*

B. Class 2 – Minor Increase

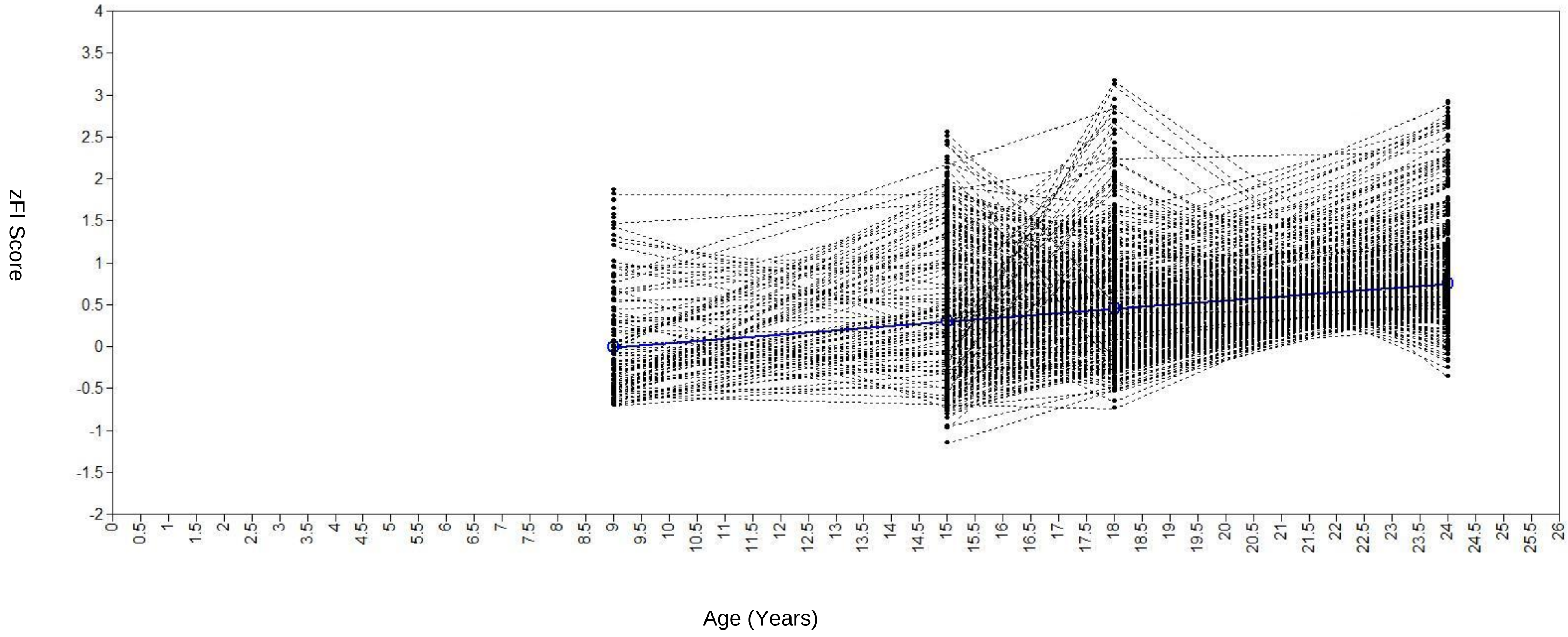

© 2021 Perry BI et al. *JAMA Psychiatry.*

C. Class 3 – Persistently High

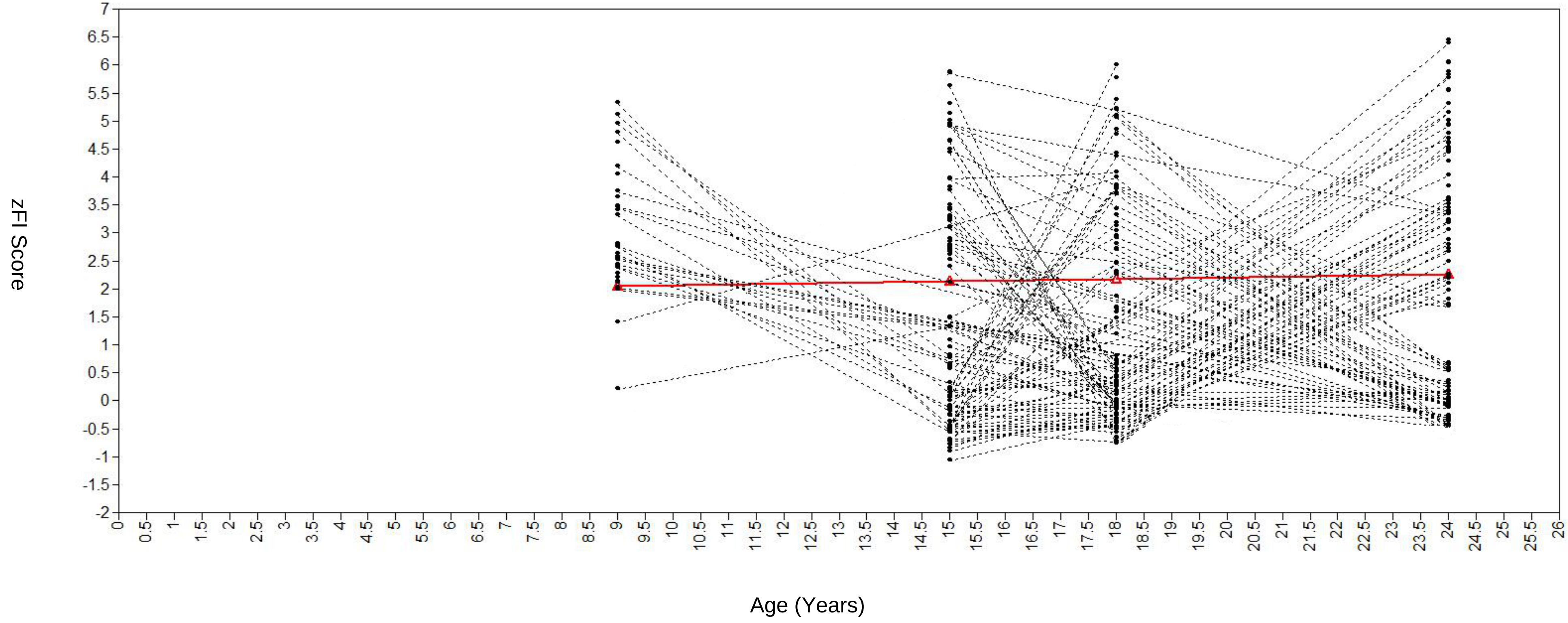

© 2021 Perry BI et al. *JAMA Psychiatry.*

eFigure 3: Sensitivity Analysis Examining Trajectories of Fasting Insulin Between Ages 15-24

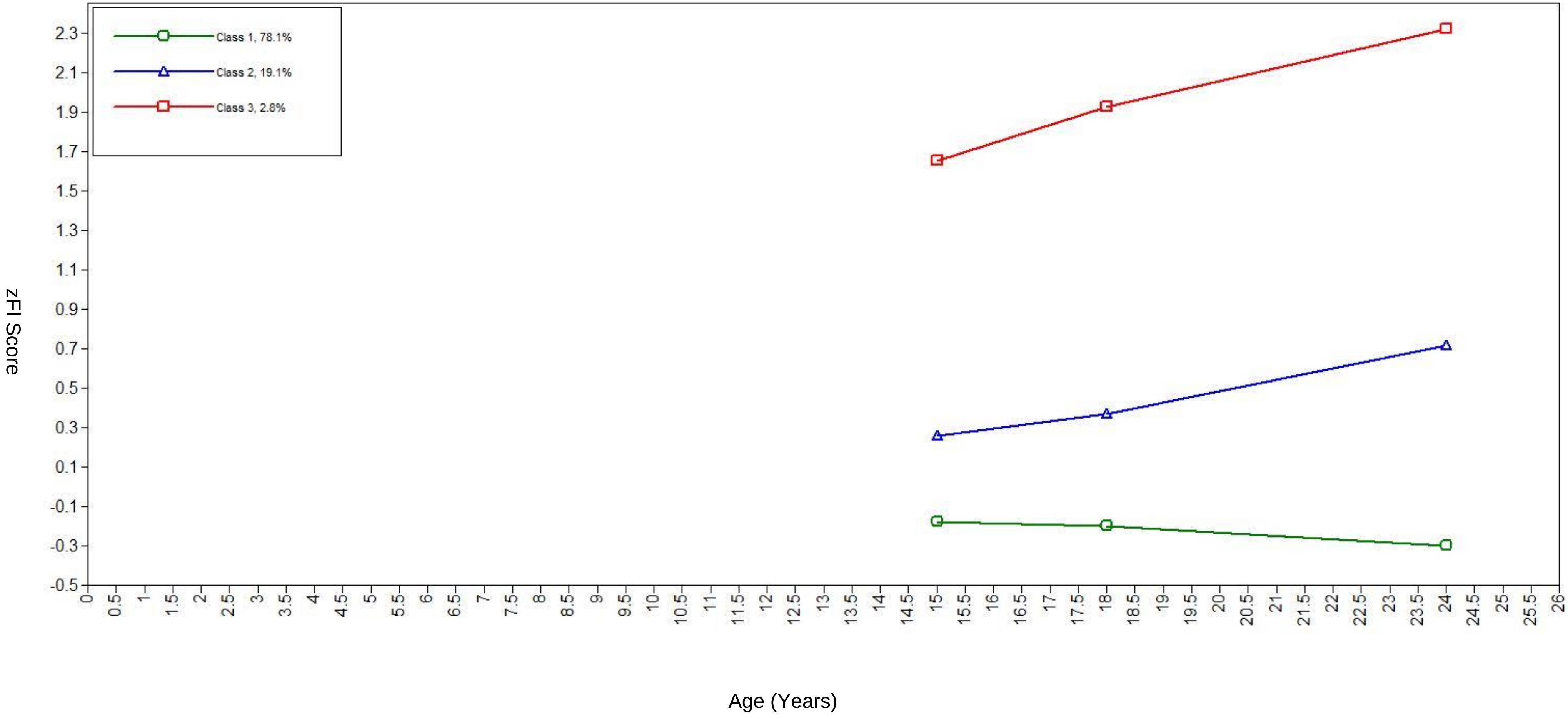

© 2021 Perry BI et al. *JAMA Psychiatry.*

eFigure 4: Trajectory Means and Individual Values per Developmental Trajectory of Body Mass Index

A. Class 1: Stable Average

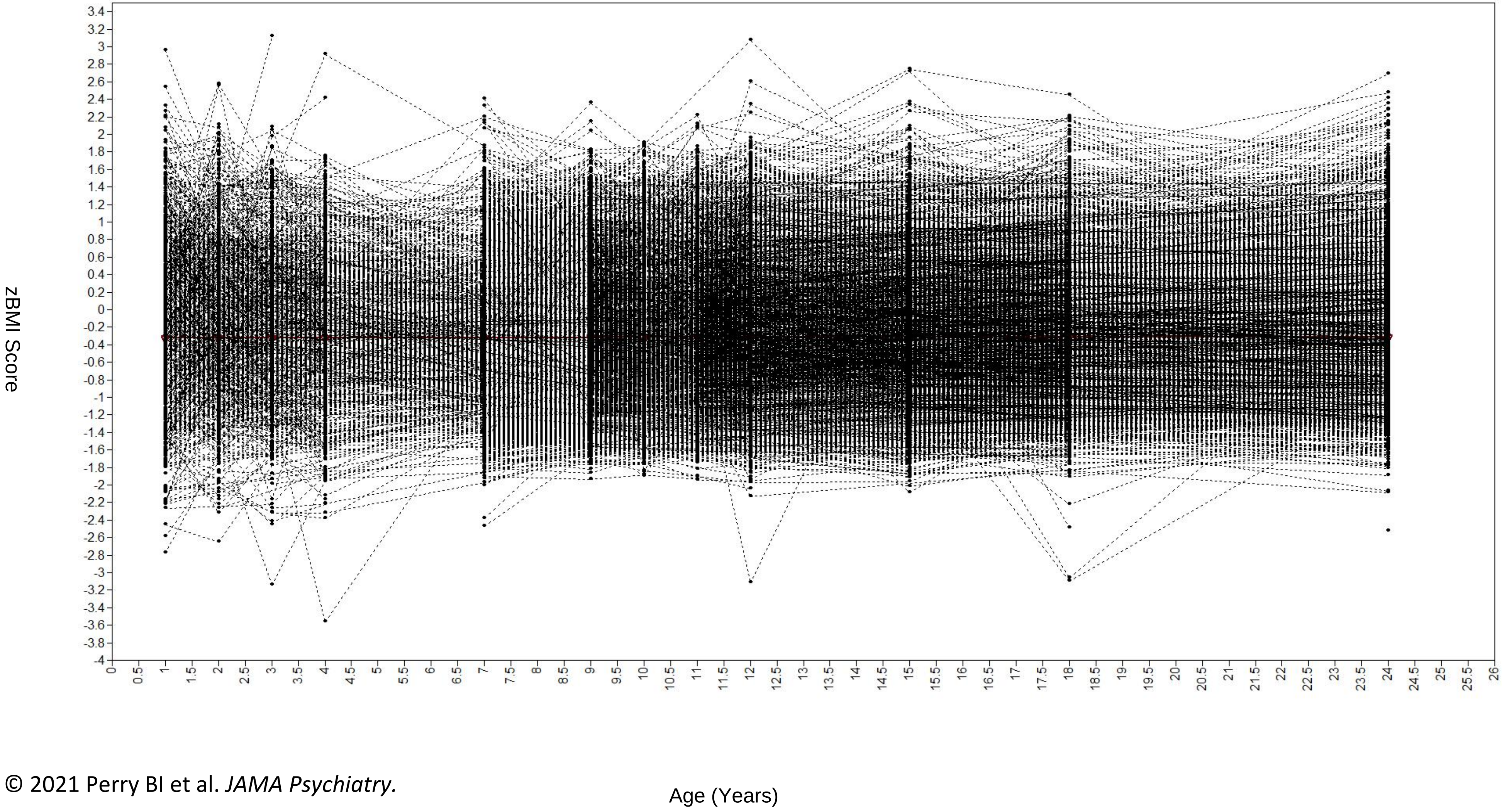

© 2021 Perry BI et al. *JAMA Psychiatry.*

B. Class 2: Gradually Decreasing

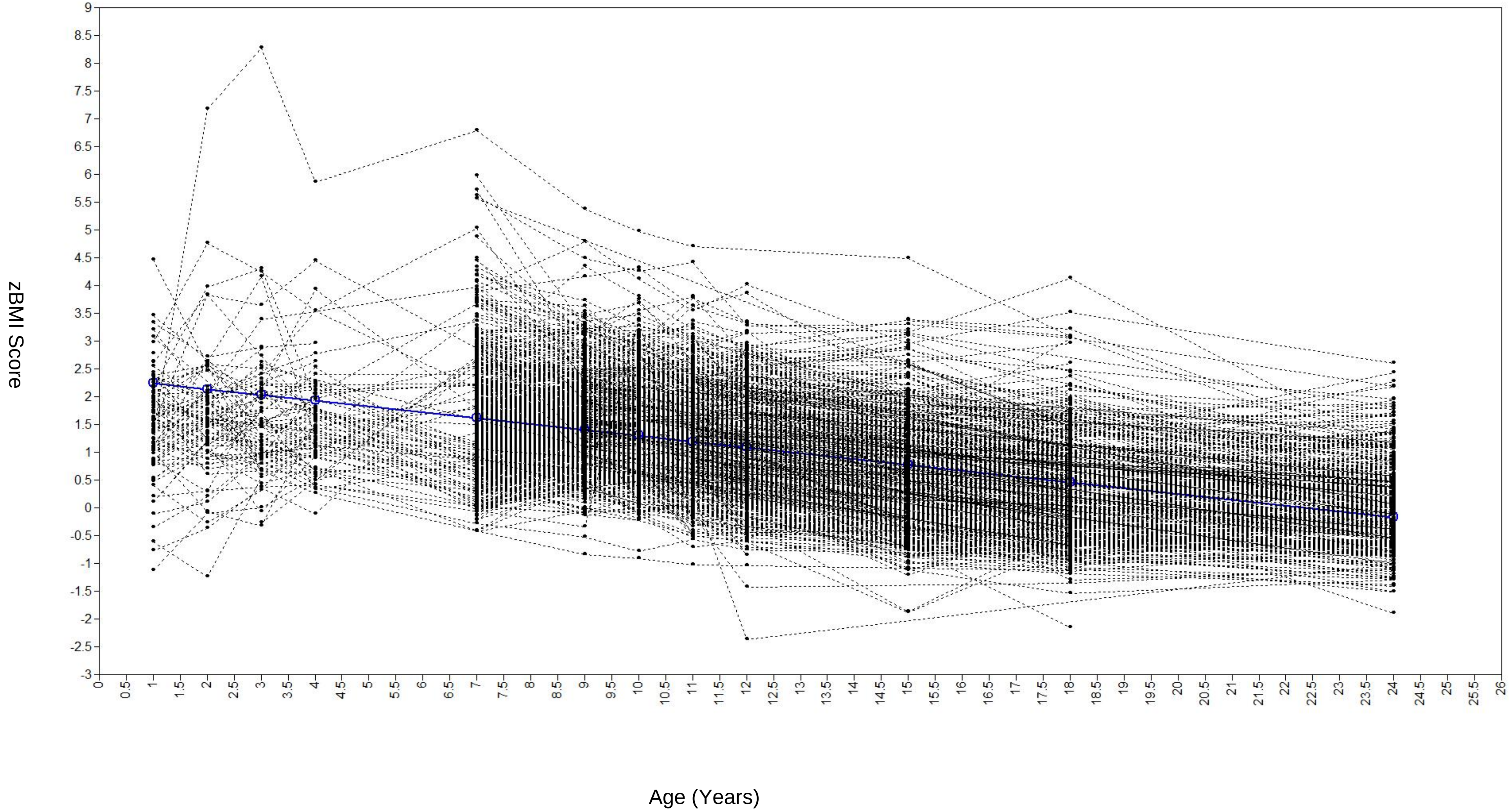

© 2021 Perry BI et al. *JAMA Psychiatry.*

C. **Class 3: Puberty Onset – Minor Increase**

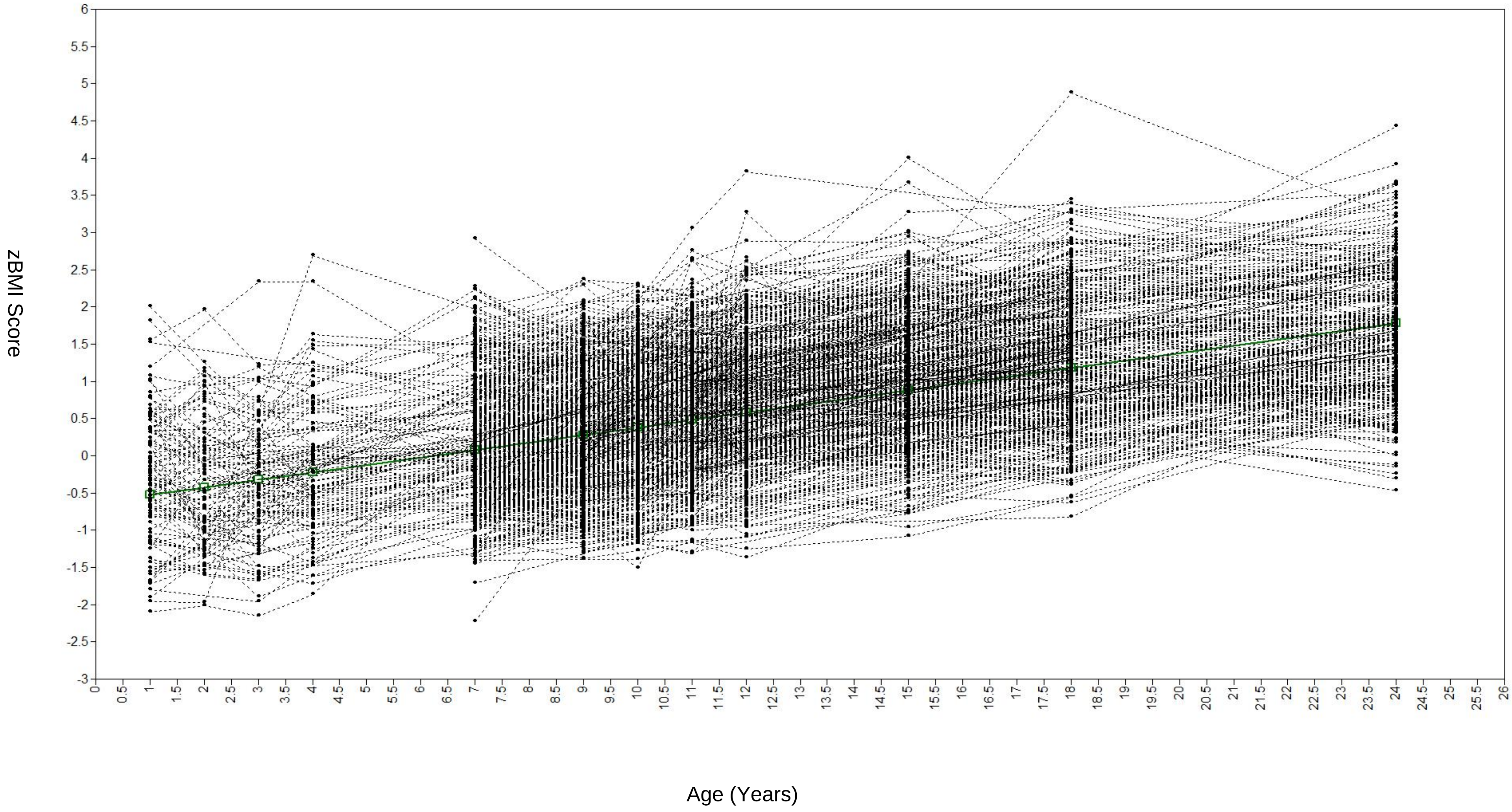

© 2021 Perry BI et al. *JAMA Psychiatry.*

D. Class 4: Puberty Onset – Major Increase (1.9% of Sample)

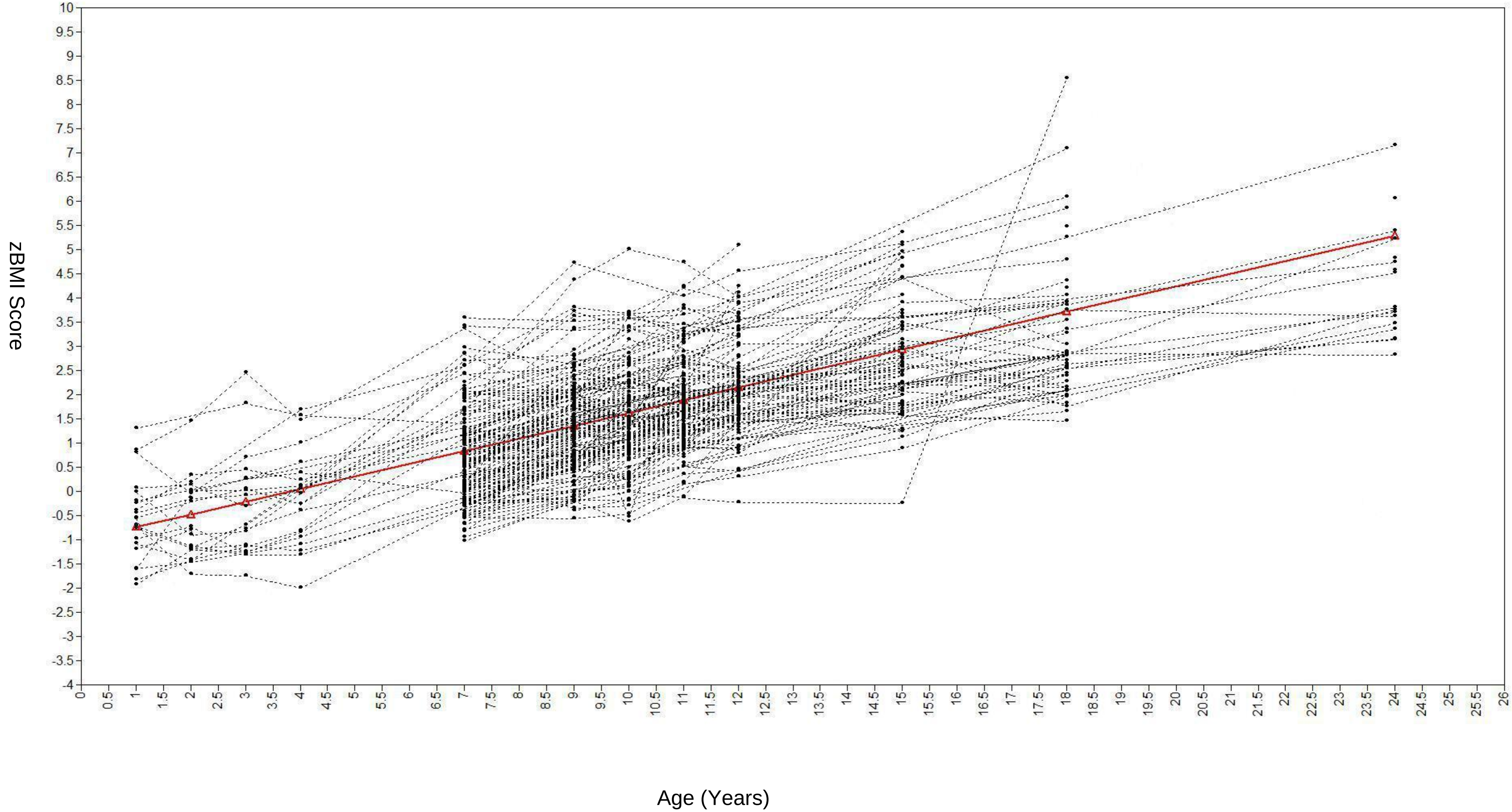

© 2021 Perry BI et al. *JAMA Psychiatry.*

E: Class 5: Persistently High

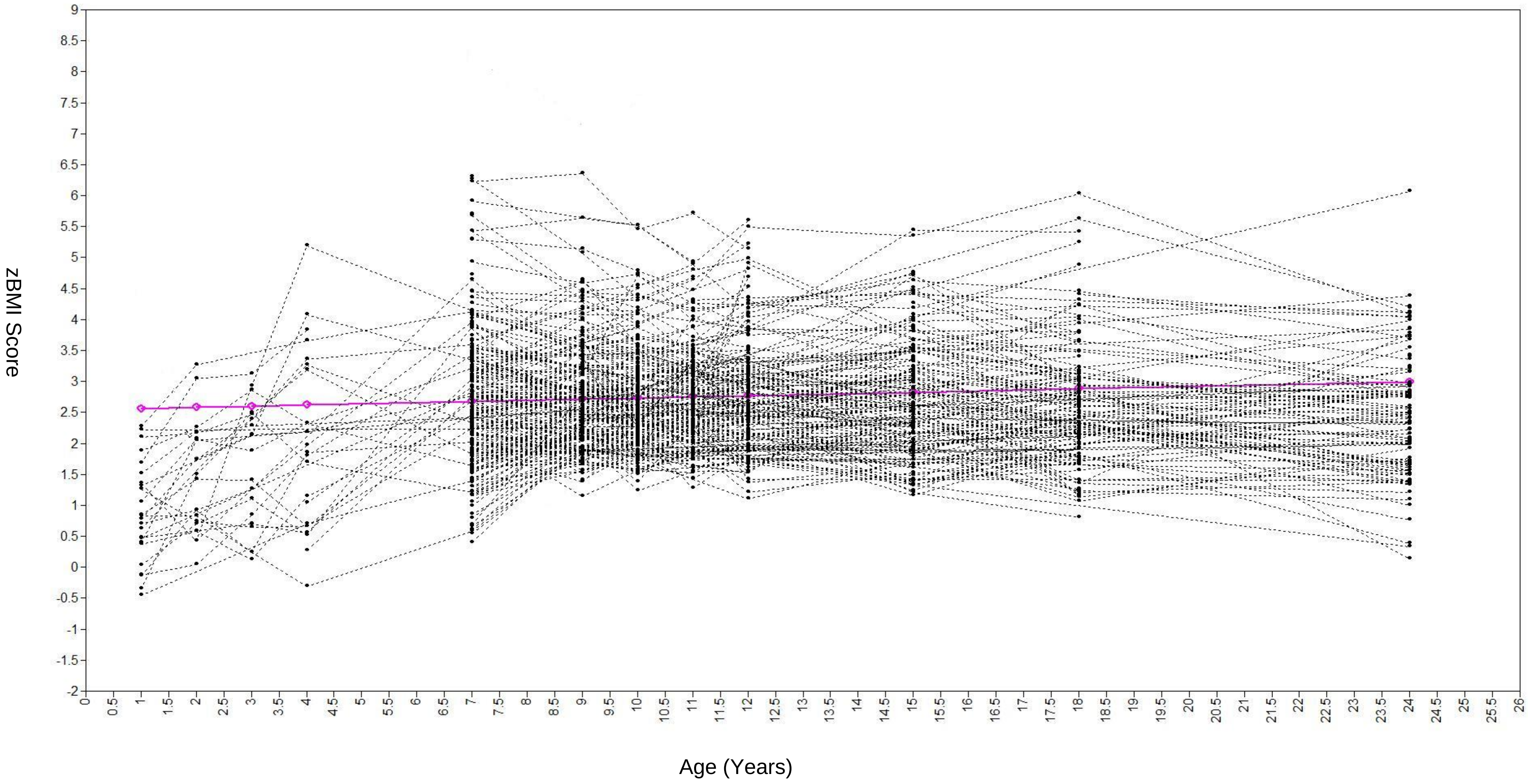

© 2021 Perry BI et al. *JAMA Psychiatry.*

© 2021 Perry BI et al. *JAMA Psychiatry.*

eReferences

1. Goodman R. Psychometric properties of the strengths and difficulties questionnaire. *J Am Acad Child Adolesc Psychiatry.* 2001;40(11):1337-1345.
2. Kowarik A, Templ, M. Imputation with the R Package VIM. *Journal of Statistical Software.* 2016;74(7).
3. Schmitt P, Mandel, J., Guedj, M. A Comparison of Six Methods for Missing Data Imputation. *Journal of Biometrics & Biostatistics.* 2015;6(224).
4. Liao SG, Lin, Y., Kang, D.D., Chandra, D., Bon, J., Kaminski, N., Sciurba, F.C., Tseng, G.C. Missing value imputation in high-dimensional phenomic data: Imputable or not, and how? *BMC Bioinformatics.* 2014;15(1):346.
5. Hallquist MN, Wiley, J.F. MplusAutomation: An R Package for Facilitating Large-Scale Latent Variable Analyses in Mplus. *Structural Equation Modeling: A Multidisciplinary Journal.* 2018;25(4):621-638.
6. Dong Y, Peng CY. Principled missing data methods for researchers. *Springerplus.* 2013;2(1):222.
7. Enders CK, Bandalos, D.L. The Relative Performance of Full Information Maximum Likelihood Estimation for Missing Data in Structural Equation Models. *Structural Equation Modeling: A Multidisciplinary Journal.* 2001;8(3):430-457.
8. Enders CK. A Primer on Maximum Likelihood Algorithms Available for Use With Missing Data. *Structural Equation Modeling: A Multidisciplinary Journal.* 2001;8(1):128-141.
9. Jung T, Wickrama, K.A.S. An Introduction to Latent Class Growth Analysis and Growth Mixture Modeling. *Social and Personality Psychology Compass.* 2007;2(1):302-317.
10. Asparouhov T, Muthen, B. Auxiliary Variables in Mixture Modeling: Three-Step Approaches Using Mplus. *Structural Equation Modeling: A Multidisciplinary Journal.* 2014;21(3):329-341.
11. Kingsbury M, Weeks M, MacKinnon N, et al. Stressful Life Events During Pregnancy and Offspring Depression: Evidence From a Prospective Cohort Study. *J Am Acad Child Adolesc Psychiatry.* 2016;55(8):709-716 e702.
12. Alberti KG, Eckel RH, Grundy SM, et al. Harmonizing the metabolic syndrome: a joint interim statement of the International Diabetes Federation Task Force on Epidemiology and Prevention; National Heart, Lung, and Blood Institute; American Heart Association; World Heart Federation; International Atherosclerosis Society; and International Association for the Study of Obesity. *Circulation.* 2009;120(16):1640-1645.

© 2021 Perry BI et al. *JAMA Psychiatry.*
